# Supplementary material for: Vector control for malaria prevention during humanitarian emergencies: protocol for a systematic review and meta-analysis
Source: BMJ Open. 2021 Jul 27;11(7):e046325. doi: 10.1136/bmjopen-2020-046325 (PMC8317076; doi:10.1136/bmjopen-2020-046325)
Supplement: Author's manuscript [file bmjopen-2020-046325.draft_revisions.pdf]

BMJ Open is committed to open peer review. As part of this commitment we make the peer review history of every article we publish publicly available.

When an article is published we post the peer reviewers' comments and the authors' responses online. We also post the versions of the paper that were used during peer review. These are the versions that the peer review comments apply to.

The versions of the paper that follow are the versions that were submitted during the peer review process. They are not the versions of record or the final published versions. They should not be cited or distributed as the published version of this manuscript.

BMJ Open is an open access journal and the full, final, typeset and author-corrected version of record of the manuscript is available on our site with no access controls, subscription charges or pay-per-view fees (http://bmjopen.bmj.com).

If you have any questions on BMJ Open's open peer review process please email info.bmjopen@bmj.com

BMJ Open

Vector control for malaria prevention during humanitarian emergencies: protocol for a systematic review and meta-analysis

Journal:	*BMJ Open*
Manuscript ID	bmjopen-2020-046325
Article Type:	Protocol
Date Submitted by the Author:	26-Oct-2020
Complete List of Authors:	Messenger, Louisa; London School of Hygiene & Tropical Medicine, Disease Control<br>Furnival-Adams, Joanna; London School of Hygiene & Tropical Medicine, Disease Control<br>Pelloquin, Bethanie; London School of Hygiene & Tropical Medicine, Disease Control<br>Rowland, Mark; London School of Hygiene & Tropical Medicine, Disease Control
Keywords:	Public health < INFECTIOUS DISEASES, ACCIDENT & EMERGENCY MEDICINE, Epidemiology < INFECTIOUS DISEASES

SCHOLARONE™
Manuscripts

I, the Submitting Author has the right to grant and does grant on behalf of all authors of the Work (as defined in the below author licence), an exclusive licence and/or a non-exclusive licence for contributions from authors who are: i) UK Crown employees; ii) where BMJ has agreed a CC-BY licence shall apply, and/or iii) in accordance with the terms applicable for US Federal Government officers or employees acting as part of their official duties; on a worldwide, perpetual, irrevocable, royalty-free basis to BMJ Publishing Group Ltd ("BMJ") its licensees and where the relevant Journal is co-owned by BMJ to the co-owners of the Journal, to publish the Work in this journal and any other BMJ products and to exploit all rights, as set out in our licence.

The Submitting Author accepts and understands that any supply made under these terms is made by BMJ to the Submitting Author unless you are acting as an employee on behalf of your employer or a postgraduate student of an affiliated institution which is paying any applicable article publishing charge ("APC") for Open Access articles. Where the Submitting Author wishes to make the Work available on an Open Access basis (and intends to pay the relevant APC), the terms of reuse of such Open Access shall be governed by a Creative Commons licence – details of these licences and which Creative Commons licence will apply to this Work are set out in our licence referred to above.

Other than as permitted in any relevant BMJ Author's Self Archiving Policies, I confirm this Work has not been accepted for publication elsewhere, is not being considered for publication elsewhere and does not duplicate material already published. I confirm all authors consent to publication of this Work and authorise the granting of this licence.

For peer review only

Vector control for malaria prevention during humanitarian emergencies: protocol for a systematic review and meta-analysis

Louisa A. Messenger[1], Joanna Furnival-Adams[1], Bethanie Pelloquin[1], Mark Rowland[1]

[1] Department of Disease Control, Faculty of Infectious Tropical Diseases, London School of Hygiene and Tropical Medicine, London, United Kingdom

Contact address: Louisa A. Messenger, Department of Disease Control, Faculty of Infectious Tropical Diseases, London School of Hygiene and Tropical Medicine, London, United Kingdom.
Louisa.messenger@lshtm.ac.uk

Abstract (300 words)

Introduction Humanitarian emergencies, of either natural or anthropogenic origins, are equivalent to major disasters, which can lead to population displacement, food insecurity and health system disruptions. Almost two-thirds of persons affected by humanitarian emergencies inhabit malaria endemic regions, particularly the World Health Organization (WHO) African Region, which currently accounts for 93% and 94% of malaria cases and deaths, respectively. As of late 2020, the United Nations Refugee Agency estimates there are globally 79.5 million forcibly displaced people, including 45.7 million internally displaced people, 26 million refugees, 4.2 million asylum-seekers and 3.6 million Venezuelans displaced abroad.

Methods and analyses A systematic review and meta-analysis will be conducted to evaluate the impact of different vector control interventions on malaria disease burden during humanitarian emergencies. Published and grey literature will be systematically retrieved from ten electronic databases and three clinical trials registries. A systematic approach to screening, reviewing and data extraction will be applied based on the Preferred Reporting Items for Systematic Reviews and Meta-Analysis (PRISMA) guidelines. Two review authors will independently assess full-text copies of potentially relevant articles based on inclusion criteria. Included studies will be assessed for risk of bias according to Cochrane and certainty of evidence using the GRADE approach. Eligible studies with reported or measurable risk ratios or odds ratios with 95% confidence intervals will be included in a meta-analysis. Sub-group analyses, including per study design, emergency phase, and primary mode of intervention, may be performed if substantial heterogeneity is encountered.

Ethics and dissemination Ethical approval is not required by the London School of Hygiene and Tropical Medicine to perform secondary analyses of existing anonymous data. Study findings will be disseminated via open-access publications in peer-reviewed journals, presentations to stakeholders and international policy makers, and will contribute to the latest WHO guidelines for malaria control during humanitarian emergencies.

PROSPERO registration number CRD42020214961.

For peer review only - http://bmjopen.bmj.com/site/about/guidelines.xhtml

Strengths and limitations of this study

- ⇒ This will be the first systematic review and meta-analysis of the impact of different vector control interventions on malaria disease burden during humanitarian emergencies, providing a comprehensive global evidence summary that can be updated as new evidence emerges.
- ⇒ Inclusion of a broad range of study types will allow the identification of studies from a diverse range of humanitarian contexts.
- ⇒ By definition, humanitarian emergencies are unpredictable and volatile and consequently these settings can be restrictive with respect to study design, limiting the number of community-level randomised control trials with epidemiological outcomes which can be considered in this review.
- ⇒ Lack of a sensitive literature search strategy may result in a large number of titles and abstracts to screen initially.

INTRODUCTION

Malaria in humanitarian emergencies

Malaria is a life-threatening parasitic disease caused by *Plasmodium* species and transmitted by female *Anopheles* mosquitoes.[1] In 2018, there were an estimated 228 million cases of malaria worldwide, including 405,000 deaths.[1]. As defined by the WHO, a humanitarian emergency is a calamitous situation in which the functioning of a community or society is severely disrupted, causing human suffering and material loss that exceeds the affected population's ability to cope using its own resources.[3] Both natural (e.g. earthquakes, floods or tsunamis) and anthropogenic (e.g. violent political conflicts) hazards can lead to humanitarian emergencies, which may involve population displacement, food insecurity and disruptions to health systems; all of these factors contribute to excess mortality and morbidity among affected communities. While natural disasters may be short-lived, complex emergencies are often protracted, characterized by having complex social, political and economic origins, breakdown of governmental authority and infrastructures and often armed political conflict and human rights abuses.[3] Globally, the United Nations Refugee Agency (UNHCR) currently estimates there are 79.5 million forcibly displaced people[4]; with almost two-thirds of persons affected by humanitarian emergencies inhabiting malaria endemic regions.[2] (Figure 1).

Figure 1. Left: Global map of internally displaced people (green) and refugee (red) populations, 2020. The UNHCR currently estimates there are 79.5 million forcibly displaced people, including 45.7 million internally displaced people, 26 million refugees, 4.2 million asylum-seekers and 3.6 million Venezuelans displaced abroad.[4] **Right:** Global map of *Plasmodium falciparum* parasite rate in children aged 2-10 years old, 2000-2017.[5]

Mass population displacement can increase the risk of epidemics and incidence of severe malaria, especially when immunologically-naive individuals with little to no prior malaria exposure move to areas of more intense transmission. These populations are rendered vulnerable to epidemic malaria by their lack of protective immunity, increased concentration of people in exposed settings, inadequate health services, breakdown of national malaria control programmes, loss or emigration of skilled healthcare workers, insufficient access to effective treatment and control measures, food scarcity, and concomitant infections, malnutrition and anaemia.[3] During humanitarian emergencies, malaria morbidity and

For peer review only - http://bmjopen.bmj.com/site/about/guidelines.xhtml

For peer review only

mortality can contribute to the breakdown of health services and local case management, which can both worsen and be a source of the emergency.

Humanitarian emergency features and settings

Humanitarian emergencies can be classified by their speed of onset, duration and setting, which all have implications for infectious disease control strategies (Figure 2). Rapid- and slow-onset emergencies describe a sudden or gradual deteriorating situation, respectively, while protracted (chronic) emergencies involve consistently high levels of humanitarian need. The criteria of the acute phase (Phase 1 and 2 in Figure 2), are elevated mortality rates, poor access to effective healthcare for the affected population, response needs that are beyond local or national capacity, and possible breakdown of normal coordination and response mechanisms. The post-acute phase (Phase 3), includes a reduction in peak mortality rates and improved access to humanitarian aid and healthcare. The recovery phase (Phase 4), is characterised by resumption of government, the withdrawal of humanitarian agencies, and introduction of development UN and NGO programmes and agencies. However, returning refugees may also re-integrate, bringing malaria with them, and depending on the level of strategic interest this phase may or may not be neglected internationally. Protracted emergencies can remain entrenched in the post-acute/early-recovery phases or cycle between acute and post-acute phases indefinitely.

Figure 2. Phases of sudden-onset, slow-onset or protracted (chronic) humanitarian emergencies. Time is indicated in days (D), weeks (W) and months (M). Adapted from [3].

Malaria vector control during humanitarian emergencies

During the acute phase of a humanitarian emergency, the first priorities for malaria control are effective case management, with prompt diagnosis and treatment.[3] Choice of vector control intervention will be determined by a number of key factors including, malaria infection risk and transmission dynamics, population demographics, prior malaria exposure, behaviour and mobility, vector species composition, vector feeding and resting preferences and emergency settings and infrastructure (Table 1).

WHO recommended malaria control interventions, insecticide-treated nets (ITNs) and indoor residual spraying (IRS), are the first line preventative measures considered. Effective case management can be supplemented with long-lasting insecticidal net (LLIN) distributions, first targeting the most vulnerable populations (e.g. pregnant women and children under-5 years old), with the goal of achieving and maintaining universal community-level coverage.[6-8] IRS can be applied in well-organised, politically-stable settings, such as transit camps, but is generally not feasible when dwellings are scattered widely, of a temporary nature, or constructed with surfaces that are unsuitable for spraying.[9] IRS is more appropriate for protecting larger refugee populations in more compact and stable settings where housing is more permanent and structurally sound, and there is an absence of alternative resting sites.[10] Targeted IRS can also supplement LLINs and be used together for insecticide resistance management.[11 12]

A suite of other community-level and personal vector control interventions has been designed for deployment during humanitarian emergencies, which will be considered in this review. One strategy has been to exploit utilitarian shelter materials, often provided in the early stages of emergencies, such as plastic sheeting, tarpaulins and tents, as mechanisms of insecticide delivery.[13-16] Additional opportunities identified to apply insecticide among at-risk populations have included distributing insecticide treated bed sheets and blankets,

For peer review only - http://bmjopen.bmj.com/site/about/guidelines.xhtml

clothing, hammocks and wall lining,[17-25] and use of topical insecticides for human populations [26 27] or to treat community livestock.[28]

Most interventions act by delivering a lethal dose of insecticide to the adult mosquito, protecting the host from exposure to a potentially infective bite. Some of these interventions, notably LLINs and IRS, may also have a community wide benefit (in areas of vector susceptibility to the active ingredients), decreasing mosquito density and longevity, and ultimately interrupting malaria transmission. The insecticide used is dependent on the intervention and resistance profiles of local vector species.[30 31] Other interventions, such as topical repellents, work by preventing mosquitoes from detecting the host, by disrupting olfactory reception.[32] These interventions only provide individual protection and do not impact vectorial capacity.[30]

There are multiple factors involved in ensuring a successful vector control programme during humanitarian emergencies. The intervention(s) selected must be feasible and consider local vector species, human resources, funding, logistics and population mobility.[3] Other benefits of vector control interventions, which may make them more acceptable to the user, include reducing flies in households and nuisance biting.[33] and improved health and yields of treated livestock.[28] Some interventions may have low acceptability, for example, sleeping under an ITN or treated blanket/bedsheet in hot, humid conditions, some types of treated clothing may be culturally inappropriate, topical repellents may cause irritation to individuals, and there may be a decrease in adherence over time to interventions which require sustained behavioural changes.[27]

Why it is important to do this review

To date, there has been insufficient, systematic syntheses of evidence for the WHO to make recommendations for deployment of malaria vector control interventions in humanitarian settings. While a number of aforementioned interventions have either been evaluated during or developed specifically for use in humanitarian emergencies, the often unpredictable and volatile nature of these settings can often be restrictive in terms of designing experimental studies. Given these logistical constraints, a range of both observational and experimental study designs will be considered.

Objectives

This study aims to evaluate the impact of different vector control interventions on malaria disease burden during humanitarian emergencies.

Primary objective is:

- To evaluate the impact of different vector control interventions on malaria disease burden during humanitarian emergencies.

Secondary objectives are:

- To explore whether the impact of the different vector control interventions varies with the phase of humanitarian emergency (acute/post-acute, protracted and recovery/post-conflict phases).
- To explore whether the impact of the different vector control interventions varies with the level of malaria transmission (low, <50% parasite rate in children; and high, >50% parasite rate in children).

For peer review only - http://bmjopen.bmj.com/site/about/guidelines.xhtml

- To explore whether the impact of the different vector control interventions varies with the level of coverage during a humanitarian emergency.

METHODS AND ANALYSES

The following types of studies for inclusion in this review:

- Cluster-randomised controlled trials (c-RCTs).
- Cluster-randomised cross-over studies.
- Cluster-randomised studies using a stepped-wedge design.
- Controlled before-and-after studies (CBAs).
- Cohort studies (prospective or retrospective).
- Non-randomised cross-over studies.
- Programmatic evaluations.
- Cross-sectional studies.
- Case-control studies.
- Case series.
- Interrupted time series (ITS).

Experimental hut trials, other phase II studies or user acceptability surveys (including, quantitative knowledge, attitude and practices (KAP) questionnaires or qualitative observations, in-depth interviews (IDIs) and focus group discussions (FGDs)) will be summarised descriptively.

Type of participants

Individuals of all age groups affected by humanitarian emergencies in malaria endemic regions; including those that have been internally displaced by humanitarian emergencies as well as host communities.

Type of interventions

Any malaria-specific vector control intervention, which has been evaluated during a humanitarian emergency, will be considered for inclusion (Table 2).

Type of outcome measures

To be eligible for inclusion, studies must report at least one of the following primary outcomes.

Primary outcomes

- Malaria case incidence: measured as number of cases per unit of time or the number of new uncomplicated malaria cases. We will use site-specific definitions as long as they have demonstrated (i) a fever or history of fever, and (ii) confirmed parasitaemia (by blood smear microscopy, rapid diagnostic test (RDT), or polymerase chain reaction (PCR)).
- Malaria infection incidence: measured as count per person per unit of time or the number of new infections (individuals must have confirmed parasitaemia by blood smear, RDT or PCR).
- Parasite prevalence (clinical and subclinical malaria): the proportion of surveyed individuals with confirmed parasitaemia at the community-level.

For peer review only - http://bmjopen.bmj.com/site/about/guidelines.xhtml

Secondary outcomes

Epidemiological

- All-case mortality.
- Severe malaria, based on confirmed malaria diagnosis (individuals must have confirmed parasitaemia by blood smear, RDT or PCR) plus the presence of clinical or laboratory features of vital organ dysfunction (these include impaired consciousness, coma, convulsions, respiratory distress, shock (systolic blood pressure <70 mmHg in adults, <50 mmHg in children), jaundice, haemoglobinuria, hypoglycaemia, severe metabolic acidosis and anaemia).
- Anaemia prevalence as per WHO cut-offs, based on haemoglobin measurements taken in community surveys.

Entomological

- Entomological inoculation rate (EIR): the estimated number of bites by infectious mosquitoes per person per unit of time. This is calculated using the human biting rate (the number of mosquitoes biting an individual over a stated period, measured directly using human baits or indirectly using light traps, knock-down catches, entry or exit traps, baited huts, or other methods of biting rate determination) multiplied by the sporozoite rate.
- Adult mosquito density: measured by a technique previously shown to be appropriate for the particular vector species (e.g. human baits, light-traps, knock-down catches, entry or exit traps, baited huts, or other methods).
- Sporozoite rate: measured as the number of caught adult mosquitoes positive for malaria sporozoites. Sporozoites can be detected through either molecular or immunological methods.

Operational

- Intervention durability: measured by a technique previously shown to be appropriate, (e.g. a time-series of WHO cone bioassays to monitor IRS residual activity or ITN/LLIN bioefficacy plus HPLC to determine intervention insecticidal content).

Adverse events

- Any indicators of adverse effects of the intervention, including the following:
  - Reports of toxicity to humans/animals
  - Environmental impacts, such as changes to the biodiversity and ecosystem due to insecticidal use
  - Changes to the levels of phenotypic/molecular insecticide resistance, assessed using standard WHO cylinder assays and/or Centers for Disease Control and Prevention (CDC) bottle bioassays/molecular techniques
  - Changes in mosquito species composition, e.g. species replacement or behaviour that reduces the efficacy of vector control interventions, e.g. an increase in exophily, exophagy, zoophily, or changes in biting time

Unintended effects

For peer review only

- Impact on human behaviour, such as changes to individuals' sleeping arrangements or use of other vector control interventions.
- Other vector-borne disease case incidence (for example, dengue and leishmaniasis).

Search methods for identification of studies

We will attempt to identify all relevant trials and studies, regardless of language, date or publication status (published, unpublished, in press and in progress).

Electronic searches

We will search the following databases: Cochrane Infectious Disease Group Specialized Register; Central Register of Controlled Trials (CENTRAL), published in the Cochrane Library; MEDLINE (OvidSP); Africa-Wide Information (EBSCO); the WHO Global Index Medicus; the Science Citation Index Expanded (Web of Science); the Conference Proceedings Citation Index – Science (Web of Science); Embase (OvidSP); Global Health (CABI); and LILACS. We will also search the WHO International Clinical Trials Registry Platform (www.who.int/ictrp/search/en), ClinicalTrials.gov (www.clinicaltrials.gov), and the ISRCTN registry (www.isrctn.com) to identify ongoing trials.

Searching other resources

We will contact past and present personnel at key stakeholders, donors, global policy makers, technical groups, non-governmental organisations (NGOs), manufacturers, implementers and researchers in the field to identify sources of unpublished data. We will also check the reference lists of studies identified by the above methods.

Data collection and analysis

Selection of studies

Study selection will follow PRISMA guidelines. Two review authors will independently assess the titles and abstracts of studies identified by the literature searches. These two review authors will assess full-text copies of potentially relevant articles based on inclusion criteria. We will compare the results of our assessments and resolve any disagreements by discussion and consensus, with arbitration by a third review author, if necessary. We will ensure that multiple publications of the same study are included once. We will list excluded studies, together with their reasons for exclusion, in a 'Characteristics of excluded studies' table; any highly relevant studies which did not meet the inclusion criteria, may be included in a separate annex. The results of the study selection process will be illustrated in a PRISMA diagram.

Data extraction and management

Two review authors will independently extract information from the included studies into a pre-piloted database, in which study settings, methods and results from identified studies will be recorded. Disagreements in data extraction will be resolved by discussion and consensus between the two review authors and arbitration by a third review author, if necessary. In case of missing data, we will contact the original study author(s) for clarification. Data will be extracted on the following:

- Lead author and publication year.
- Study period.

For peer review only - http://bmjopen.bmj.com/site/about/guidelines.xhtml

- Study design: type of study; method of participant selection (for c-RCTs: adjustment for clustering); cluster size; number of clusters; cross over; sample size.
- Study participants: population size; origins and length of time in setting (with respect to the particular emergency situation); characteristics: age, sex, ethnicity, recruitment rates, withdrawal, loss to follow up, socioeconomic status; levels of prior malaria exposure; proportion of vulnerable individuals; prevalence of co-morbidities.
- Humanitarian emergency context: geographic country or area affected; underlying cause(s) of emergency and relevant mitigating factors; structure and layout of residence area (e.g. formal camp, informal settlement, open/closed); type of accommodation or housing available in study area; displacement dynamics and mobility; road access and condition; details of the political and logistical constraints encountered during different phases of humanitarian emergencies and their impact on the feasibility and choice of vector control intervention(s).
- Epidemiological context: malaria transmission intensity; incidence; prevalence; *Plasmodium* species.
- Intervention: description of intervention (active ingredient, dose, formulation, intervention format, frequency and timing of implementation); coverage, usage and adherence; any reports of refusals and reasons why.
- Cointerventions: details of any other malaria or vector-specific control interventions (such as, mass drug administration, active case detection, health education, untreated bednets); coverage and adherence; any reports of refusals and reasons why.
- Delivery mechanism(s): route of intervention deployment (for example, community-wide dissemination, health facility-based distributions, integration with other essential services); any additional information related to the method of implementation, including who implemented the intervention and who was targeted (the general population or a sub-population).
- Outcome measurements: definition of outcome; diagnostic or surveillance method; passive or active case detection; duration of follow-up; time points at which outcomes were assessed; number of events; number of participants or unit time; statistical power; unit of analysis; incomplete outcomes/missing data.
- Other: primary and secondary vector(s) species; vector(s) behaviour (population seasonality, adult habitat, peaking biting times, exophilic/endophilic, exophagic/endophagic, anthropophilic/zoophilic); method of vector collection(s); baseline measures of phenotypic insecticide resistance and any measures during the study period; baseline measures of genotypic insecticide resistance and any measure during the study period; any measure of vector sporozoite rate; identification of vector bloodmeal(s); vector parity and age structure; any measure of user acceptability collected during the trial and reported by the treatment arm, including cross-sectional survey data of reported acceptability and qualitative data on opinions about the intervention.
- Identification of major limitations in design, analysis, results, and any aspects that would limit generalizability of results.
- Source of funding of the study; reported conflicts of interest of authors.

For dichotomous outcomes, we will extract the number of participants experiencing each outcome and number of participants in each study group. For continuous outcomes, we will extract number of participants in each study group, the mean and a measure of variance (for example, standard deviation or standard error). For count/rate data outcomes, we will extract the number of outcomes in the treatment and control groups, and the total person time at risk in each group (or the rate ratio), and a measure of variance. For c-RCTs, we will record

For peer review only

For peer review only - http://bmjopen.bmj.com/site/about/guidelines.xhtml

the number of clusters randomised, number of clusters analysed, measure of effect e.g. risk ratio, odds ratio or mean difference, with confidence intervals (CIs) or standard deviations, number of participants and the intra-cluster correlation coefficient (ICC) value. For non-randomised studies, we will extract adjusted measures of effects that attempt to control for confounding. If there are no adjusted measures reported, then we will extract unadjusted measures.

Depending on the number of eligible studies per vector control strategy, interventions may be grouped together and analysed according to their primary mode of action against vector populations. Studies may be classified as:

1) Community-wide insecticidal effect, primarily targeting indoor resting vectors, e.g. IRS, insecticide-treated temporary housing materials and wall linings.
2) Personal protective insecticidal effect, primarily targeting indoor feeding vectors, e.g. insecticide-treated bedding material and clothing.
3) Personal protective insecticidal effect, primarily targeting outdoor feeding vectors, e.g. personal repellents.
4) Dual community-wide and personal protective insecticidal effects, primarily targeting indoor feeding and resting vectors, e.g. ITNs/LLINs and insecticide-treated curtains.
5) Community-wide insecticidal effect, primarily targeting outdoor resting and feeding vectors, e.g. insecticide treatment of livestock.
6) Community-wide insecticidal effect, targeting immature vector stages and potential breeding sites, e.g. LSM.

Assessment of risk of bias in included studies

Two review authors will independently assess the risk of bias for each study, consulting a third review author if necessary. For c-RCTs, we will use the Cochrane ‘Risk of bias’ tool, as well as the six additional criteria listed in Section 23.1.2 of the *Cochrane Handbook for Systematic Reviews of Interventions* that relate specifically to c-RCTs.[34] For randomised cross-over trials, we will use the Cochrane 'Risk of bias' tool, as well as the additional criteria listed in Section 23.2.3 of the *Cochrane Handbook for Systematic Reviews of Interventions* that relate specifically to randomised cross-over trials. For non-randomised controlled studies, we will use the Cochrane Effective Practice and Organisation of Care (EPOC) 'Risk of bias' tool. Biases will be classified as ‘low’, ‘high’ or ‘unclear’ and summarised in graphs. For observational studies, we will use the Cochrane Risk Of Bias In Non-randomised Studies - of Interventions (ROBINS-I) tool.[35] We will assess risk of bias through a hierarchy of domains, starting with ‘critical’, then ‘serious’, ‘moderate’, and ‘low’. If any domain reaches critical risk of bias we will not continue with the assessment. We will make a risk of bias assessment for each outcome. If risk of bias is critical it will be excluded from the review. Highly relevant studies with a critical risk of bias may be included in a separate annex.

Measures of treatment effect

We will compare intervention and control data using (preferably) risk ratios or odds ratios if the outcome is dichotomous. For continuous data, we will present the mean difference (when studies use the same outcome measurement scale, and SMD when studies use different outcome measurement scale); and for count/rate data, we will use rate ratios. We will use adjusted measures of effect to summarise treatment effects from non-randomised studies. We will present all results with their associated 95% CIs. We will report any accounts that signal adverse effects.

For peer review only - http://bmjopen.bmj.com/site/about/guidelines.xhtml

Unit of analysis issues

For c-RCTs, or non-randomised cluster trials, we will extract adjusted measures of effect where possible. If the study authors did not perform any adjustment for clustering, we will adjust the raw data ourselves using an ICC value. If an ICC is not reported, we will estimate this, with reference to similar studies, if possible. We will not present results from c-RCTs that are not adjusted for clustering. If we estimate the ICC, we will perform sensitivity analyses to investigate the robustness of our analyses. If we identify studies for inclusion that have multiple intervention arms, we will include data from these studies by either combining treatment arms, or by splitting the control group so that we only include these participants in the meta-analysis once. We will consider the level at which randomization occurred, such as cross-over trials, c-RCTs, and multiple observations for the same outcome. For randomised cross-over trials, where neither carry-over nor period effects are identified as problematic, we will use a paired t-test for the analysis of continuous data from two-period, two-armed cross-over trials.

Dealing with missing data

In case of missing data, we will apply available-case analysis and will include data on known results, as well as 'non-adherent' participants, excluded from the primary study authors' analysis, but for whom data are available.[36] 'Non-adherent' participant data may be included in "intention-to-treat" or "as-treated" analyses, as appropriate.[36] The denominator will vary according to the total number of participants who had data recorded for the specific outcome as well as the total number of excluded participants included per analysis type. For outcomes with no missing data, we plan to perform analyses on an "intention-to-treat" basis. We will include all participants randomised to each group in the analyses and will analyse participants in the group to which they were randomised. If further data are needed, respective authors, companies or organisations will be contacted to obtain missing data.

Assessment of heterogeneity

Heterogeneity will be assessed by visually examining the overlap of study CIs, applying Chi^2 and I^2 statistics. A Chi^2 test result with P value < 0.1 will indicate significant heterogeneity. Interpretation of I^2 will follow the classification: 30% to 60%: may represent moderate heterogeneity; 50% to 90%: substantial heterogeneity; 75% to 100%: considerable heterogeneity. If we observe substantial heterogeneity we will perform further subgroup analysis. If appropriate we will explore clinical and methodological heterogeneity through consideration of the trial populations, methods, and interventions and by visualization of trial results.

Assessment of reporting biases

We will construct a funnel plot to assess reporting biases when performing an analysis of 10 or more studies. We will explore reasons for funnel plot asymmetry, including true heterogeneity of the effect with respect to trial size, poor methodological design (and hence bias of small trials), and publication bias, and will interpret results accordingly.

Data synthesis

We will analyse data using Review Manager 5.[37] We will use fixed-effect meta-analysis to combine data if heterogeneity is absent. If considerable heterogeneity is present, we will combine data using random-effects meta-analysis and report an average treatment effect.

For peer review only - http://bmjopen.bmj.com/site/about/guidelines.xhtml

We will decide whether to use fixed-effect or random-effects models based on the consideration of clinical and methodological heterogeneity between trials.

Certainty of the evidence

We will assess the certainty of evidence using the GRADE approach.[38] Evidence will start as high-certainty. However, if there is bias due to confounding, missing data or selection of the report result, outcomes will be marked as moderate, high or unclear risk of bias and the evidence will be rated down accordingly to low-certainty evidence.

For c-RCTs, we will rate each important outcome as described by.[39] c-RCTs start as high quality evidence but can be downgraded if there are valid reasons within the following five categories: risk of bias, imprecision, inconsistency, indirectness, and publication bias. Non-randomized studies can also be upgraded if there is a large effect, and if all plausible residual confounding would reduce a demonstrated effect or would suggest a spurious effect if no effect was observed.[39] We will summarise our findings in a 'Summary of findings' table.

We will summarise qualitative findings on consumer views e.g. user acceptability narratively. If there are a sufficient number of included studies, two review authors will independently code the studies, and use thematic synthesis to identify themes and subthemes.

Subgroup analysis and investigation of heterogeneity

We will stratify the analysis by study design, phase of humanitarian emergency (acute/post-acute, protracted and recovery/post-conflict phases) and primary mode of intervention action. Where substantial heterogeneity exists, we plan to perform the following subgroup analysis:

- Intervention coverage (appropriate levels to be determined with respect to the primary mode of action of each intervention, e.g. community-wide mass effect *vs.* personal protection).
- Participants (<5 years, pregnant women, adult, mixed age groups).
- Geographical region.
- Malaria vector species behaviour.
- Malaria transmission dynamics/seasonality.

Sensitivity analysis

We will perform sensitivity analysis on the primary outcome, to observe the effect of excluding trials at high risk of bias (for incomplete outcome data). If the ICC value is estimated, we will undertake sensitivity analyses to investigate the impact of varying the ICC value on meta-analysis results.

ETHICS AND DISSEMINATION

This systematic review and meta-analysis will consist of secondary analyses of existing anonymous data and meets the criteria for waiver of ethics review by the London School of Hygiene and Tropical Medicine. As this research is based on previously published data, there will be no patient and public involvement in the design, interpretation or dissemination

For peer review only

of study findings. This study protocol was drafted according to the Preferred Reporting Items for Systematic Reviews and Meta-Analyses Protocols (PRISMA-P) checklist. [40] Any amendments to this protocol will be documented and published alongside the results of the systematic review and meta-analysis. Study findings will be disseminated via open-access publications in peer-reviewed journals, presentations to stakeholders and relevant national and international policy makers and will contribute to the latest WHO guidelines for malaria control during humanitarian emergencies.

Authors' contributions

LAM, JFA, BP and MR wrote the full text of the protocol. All review authors read and approved the final protocol draft.

Funding statement

This work was supported by the World Health Organization Global Malaria Programme (WHO-GMP). LAM, JFA and MR are supported by the DFID/MRC/NIHR/Wellcome Trust Joint Global Health Trials Scheme. LAM and MR are also supported by UNITAID/Global Fund and DFID-RPC. BP is supported by the LSHTM-Nagasaki University Joint PhD Programme for Global Health. We are very grateful to Jane Falconer, Information Specialist, at the London School of Hygiene and Tropical Medicine, for help with the literature search strategy, and to Drs. Jan Kolaczinski, Jennifer Stevenson and Elie Akl (WHO-GMP) and Drs. Natacha Protopopoff and Natasha Howard (LSHTM) for valuable protocol suggestions. The funder of the study had no role in study design, data collection, data analysis, data interpretation, or writing of the report. The corresponding author had full access to all the data in the study and had final responsibility for the decision to submit for publication.

Competing interests statement.

The authors declare no competing interests.

References

1. World Health Organization 2019. World Malaria Report. Geneva, Switzerland: World Health Organization 2019.
2. Anderson J, Doocy S, Haskew C, *et al.* The burden of malaria in post-emergency refugee sites: a retrospective study. *Confl Health* 2011;5:17.
3. World Health Organization 2013. Malaria control in humanitarian emergencies: an inter-agency field handbook. Second edition Geneva: 2013.
4. The UN refugee Agency (UNHCR), 2020. Figures at a glance. Accessible from: https://www.unhcr.org/uk/figures-at-a-glance.html Accessed September 5th 2020.
5. The Malaria Atlas Project (MAP), 2020. Interactive map. Accessible from: https://malariaatlas.org/ Accessed September 5th 2020.
6. Spencer S, Grant AD, Piola P, *et al.* Malaria in camps for internally-displaced persons in Uganda: evaluation of an insecticide-treated bednet distribution programme. *Trans R Soc Trop Med Hyg* 2004;98(12):719-27.
7. Richards AK, Banek K, Mullany LC, *et al.* Cross-border malaria control for internally displaced persons: observational results from a pilot programme in Eastern Burma/Myanmar. *Trop Med Int* Health 2009;14(5):512-21.

For peer review only - http://bmjopen.bmj.com/site/about/guidelines.xhtml

8. Smithuis FM, Kyaw MK, Phe UO, *et al.* The effect of insecticide-treated bed nets on the incidence and prevalence of malaria in children in an area of unstable seasonal transmission in western Myanmar. *Malaria J* 2013;12:363.
9. Charlwood JD, Qassim M, Elnsur EI, *et al.* The impact of indoor residual spraying with malathion on malaria in refugee camps in eastern Sudan. *Acta Trop* 2001;80(1):1-8.
10. Rowland M, Nosten F. Malaria epidemiology and control in refugee camps and complex emergencies. *Ann Trop Med Parasit* 2001;95(8):741-54.
11. Rowland, M, Hewitt S, Durrani N, *et al.* Sustainability of pyrethroid-impregnated bednets for malaria control in Afghan communities. *Bull World Health Organ* 1997;75(1):23-29.
12. World Health Organization 2012. Global plan for insecticide resistance management in malaria vectors. Geneva, Switzerland: World Health Organization 2012.
13. Bouma MJ, Parvez SD, Nesbit R, *et al.* Malaria control using permethrin applied to tents of nomadic Afghan refugees in northern Pakistan. *Bull World Health Organ* 1996;74(4):413-21.
14. Graham K, Mohammad N, Rehman H, *et al.* Insecticide-treated plastic tarpaulins for control of malaria vectors in refugee camps. *Med Vet Entomol* 2002;16:404-08.
15. Graham K, Rehman H, Ahmad M, *et al.* Tents pre-treated with insecticide for malaria control in refugee camps: an entomological evaluation. *Malaria J* 2004;3:25.
16. Burns M, Rowland M, N'Guessan R, *et al.* Insecticide-treated plastic sheeting for emergency malaria prevention and shelter among displaced populations: an observational cohort study in a refugee setting in Sierra Leone. *Am J Trop Med Hyg* 2012;87(2):242-50.
17. Graham K, Mohammad N, Rehman H, *et al.* Comparison of three pyrethroid treatments of top-sheets for malaria control in emergencies: entomological and user acceptance studies in an Afghan refugee camp in Pakistan. *Med Vet Entomol* 2002;16:199-206.
18. Rowland M, Durrani N, Hewitt S, *et al.* Permethrin-treated chaddars and top-sheets: appropriate technology for protection against malaria in Afghanistan and other complex emergencies. *Trans R Soc Trop Med Hyg* 1999;93(5):465-72.
19. Macintyre K, Sosler S, Letipila F, *et al.* A new tool for malaria prevention?: results of a trial of permethrin-impregnated bedsheets (*shukas*) in an area of unstable transmission. *Int J Epidemiol* 2002;32:157-60.
20. Kimani EW, Vulule JM, Kuria IW, *et al.* Use of insecticide-treated clothes for personal protection against malaria: a community trial. *Malaria J* 2006;5:63.
21. Sochantha T, Van Bortel W, Savonnaroth S, *et al.* Personal protection by long-lasting insecticidal hammocks against the bites of forest malaria vectors. *Trop Med Int Health* 2010;15(3):336-41.
22. Sharma SK, Upadhyay AK, Haque MA, *et al.* Field evaluation of ZeroFly--an insecticide incorporated plastic sheeting against malaria vectors and its impact on malaria transmission in tribal areas of northern Orissa. *Indian J Med Res* 2009;130(4):458-66.
23. Messenger LA, Miller NP, Adeogun AO, *et al.* The development of insecticide-treated durable wall lining for malaria control: insights from rural and urban populations in Angola and Nigeria. *Malaria J* 2012;11:332.
24. Kitau J, Oxborough R, Kaye A, *et al.* Laboratory and experimental hut evaluation of a long-lasting insecticide treated blanket for protection against mosquitoes. *Parasit Vectors* 2014;7:129.
25. Reyburn H, Ashford R, Mohsen M, *et al.* A randomised controlled trial of insecticide-treated bednets and chaddars or top-sheets, and residual spraying of interior rooms for the prevention of cutaneous leishmaniasis in Kabul, Afghanistan. *Trans R Soc Trop Med Hyg* 2000;94:361-366.

For peer review only

For peer review only - http://bmjopen.bmj.com/site/about/guidelines.xhtml

26. Rowland M, Downey G, Rab A, *et al.* DEET mosquito repellent provides personal protection against malaria: a household randomized trial in an Afghan refugee camp in Pakistan. *Trop Med Int Health* 2004;9(3):335-42.
27. Rowland M, Freeman T, Downey G, *et al.* DEET mosquito repellent sold through social marketing provides personal protection against malaria in an area of all-night mosquito biting and partial coverage of insecticide-treated nets: a case-control study of effectiveness. *Trop Med Int Health* 2004;9(3):343-50.
28. Rowland M, Durrani N, Kenward M, *et al.* Control of malaria in Pakistan by applying deltamethrin insecticide to cattle: a community-randomised trial. *Lancet* 2001;357(9271):1837-41.
29. Majambere S, Pinder M, Fillinger U, *et al.* Is mosquito larval source management appropriate for reducing malaria in areas of extensive flooding in The Gambia? A cross-over intervention trial. *Am J Trop Med Hyg* 2012;82(2):176-84.
30. World Health Organization 2019.Global Malaria Programme. Guidelines for Malaria Vector Control.
31. World Health Organization 2020. List of WHO prequalified vector control products www.who.int/pq-vector-control/prequalified-lists/PrequalifiedProducts27January2020.pdf?ua=1. Accessed June 17th 2020.
32. Maia MF, Kliner M, Richardson M, *et al.* Mosquito repellents for malaria prevention. *Cochrane Database Syst Rev* 2018;2.
33. Furnival-Adams J, Olanga EA, Napier M, *et al.* Housing interventions for preventing malaria. *Cochrane Database Syst Rev* 2019;8.
34. Higgins JPT, Thomas J, Chandler J, *et al.* eds. *Cochrane Handbook for Systematic Reviews of Interventions* version 6.0 (updated July 2019). Cochrane, 2019. Available from www.training.cochrane.org/handbook.
35. Sterne J, Hernán MA, Reeves Barnaby C, *et al.* ROBINS-I: a tool for assessing risk of bias in non-randomised studies of interventions; *BMJ* 2016; 355; i4919.
36. Akl EA, Johnston BC, Alonso-Coello P, *et al.* Addressing dichotomous data for participants excluded from trial analysis: a guide for systematic reviewers. *PLoS One* 2013;8(2):e57132.
37. Nordic Cochrane Centre, The Cochrane Collaboration. Review Manager 5 (RevMan 5). Version 5.3. Copenhagen: Nordic Cochrane Centre, The Cochrane Collaboration, 2014.
38. Guyatt GH, Oxman AD, Schunemann HJ, *et al.* GRADE guidelines: a new series of articles in the Journal of Clinical Epidemiology. *J Clin Epidemiol* 2011;64(4):380-2.
39. Balshem H, Helfand M, Schunemann HJ, *et al.* GRADE guidelines: 3. Rating the quality of evidence. *J Clin Epidemiol* 2011;64(4):401-6.
40. Shamseer L, Moher D, Clarke M, *et al.* Preferred reporting items for systematic review and meta-analysis protocols (PRISMA-P) 2015: elaboration and explanation. *BMJ* 2015;349:g7647.

For peer review only - http://bmjopen.bmj.com/site/about/guidelines.xhtml

Table 1. Key factors influencing choice of vector control intervention(s) during humanitarian emergencies.

Key Factors	Sub-Factors
Malaria epidemiology	Level of endemicity; local transmission dynamics and stability; circulating *Plasmodium* species and proportions of co-infection; level of drug resistance; asymptomatic infections
Human population composition and behaviour	Population origin and size; displacement dynamics and mobility; level of prior malaria exposure; proportion of vulnerable individuals (for example, pregnant women, children and the elderly); age and sex distributions; sleeping location and night-time behaviour; knowledge of malaria; education; healthcare and treatment seeking behaviour; prevalence of comorbidities (for example, malnutrition, anaemia, HIV); gender mobility and work practices
Vector species distribution and behaviour	Local vector species composition; vector seasonality; host preference (anthropophillic/zoophillic); feeding time and location (early/late biting and indoor/outdoor biting); resting location (indoor/outdoor resting); level of insecticide resistance; flight range; breeding site preference
Emergency setting and infrastructure	Type of shelter or housing available (for example, *ad-hoc* refuse materials, plastic sheeting, tents, more permanent housing); road access and condition; camp or community organisation and layout; prior and present vector control interventions or programmes; timing of rainy seasons; potential to impact other vector-borne diseases (such as in areas of sympatric leishmaniasis) and nuisance insects (for example, lice, fleas, ticks, biting flies, bed bugs)

For peer review only - http://bmjopen.bmj.com/site/about/guidelines.xhtml

Table 2. Types of intervention to be included in this review.

Intervention Category	Examples of Intervention	Control*
Insecticide-treated temporary housing materials	Plastic sheeting; tarpaulins; tents	Untreated temporary housing material
Insecticide-treated bedding materials	Sheets; blankets; chaddars; top sheets	Untreated bedding material
Insecticide-treated wall linings	Plastic woven or non-woven mesh material used to cover housing structure/shelters	Untreated wall lining
Insecticide-treated clothing	Military uniforms; occupational clothing	Untreated clothing
Insecticide-treated curtains	Netted curtain or window material	Untreated curtains
Insecticide treatment of livestock	Cattle dipping; use of endectocides	No cattle dipping/endectocide use
Personal repellents	Spatial repellents; topical repellents; insecticidal soaps	No spatial repellent
Bed nets	Long-lasting insecticidal nets (LLINs); insecticide-treated nets (ITNs); untreated nets (UTNs)	Untreated bed nets (when compared to insecticidal counterparts)
Indoor residual spraying (IRS)	Chemical treatment of housing structure/shelters	No chemical treatment of housing structures/shelters
Larval source management (LSM)	Chemical or biological treatment of breeding sites; habitat modification or manipulation	No insecticidal or mechanical control

*Studies where the control group comprises no other insecticidal malaria vector control intervention will also be considered. Use of other vector control tools (in addition to the intervention under evaluation) must be balanced between study arms.

For peer review only - http://bmjopen.bmj.com/site/about/guidelines.xhtml

For peer review only

For peer review only - http://bmjopen.bmj.com/site/about/guidelines.xhtml

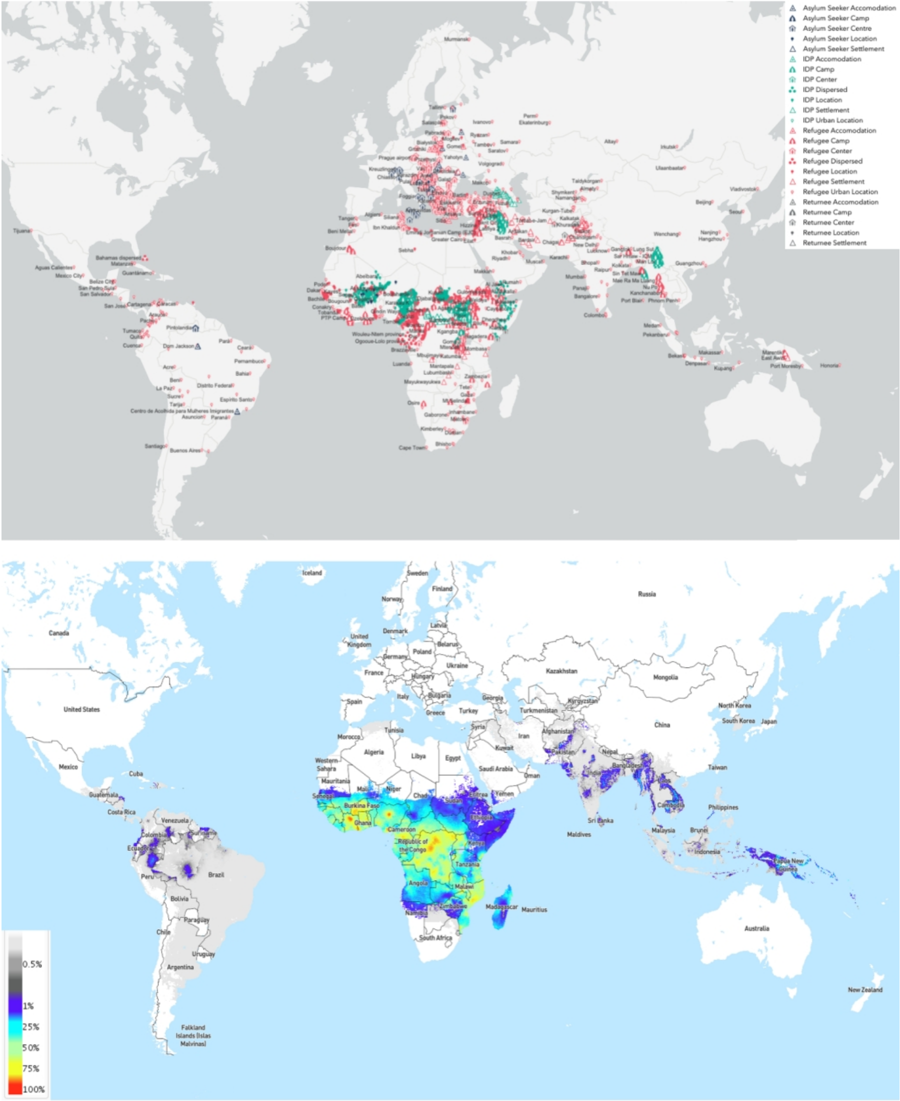

Figure 1. Left: Global map of internally displaced people (green) and refugee (red) populations, 2020. The UNHCR currently estimates there are 79.5 million forcibly displaced people, including 45.7 million internally displaced people, 26 million refugees, 4.2 million asylum-seekers and 3.6 million Venezuelans displaced abroad. Right: Global map of Plasmodium falciparum parasite rate in children aged 2-10 years old, 2000-2017.

299x400mm (127 x 127 DPI)

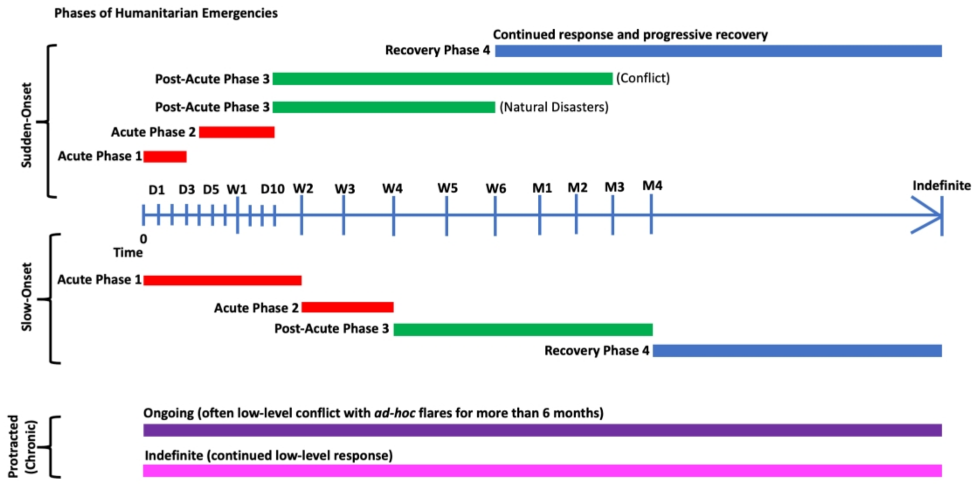

Figure 2. Phases of sudden-onset, slow-onset or protracted (chronic) humanitarian emergencies. Time is indicated in days (D), weeks (W) and months (M). Adapted from 3.

338x190mm (150 x 150 DPI)

PRISMA-P 2015 Checklist

This checklist has been adapted for use with protocol submissions to *Systematic Reviews* from Table 3 in Moher D et al: Preferred reporting items for systematic review and meta-analysis protocols (PRISMA-P) 2015 statement. *Systematic Reviews* 2015 **4**:1

Section/topic	#	Checklist item	Information reported: Yes	Information reported: No	Line number(s)
**ADMINISTRATIVE INFORMATION**					
**Title**					
Identification	1a	Identify the report as a protocol of a systematic review	X		1-2
Update	1b	If the protocol is for an update of a previous systematic review, identify as such		X	
**Registration**	2	If registered, provide the name of the registry (e.g., PROSPERO) and registration number in the Abstract	X		42
**Authors**					
Contact	3a	Provide name, institutional affiliation, and e-mail address of all protocol authors; provide physical mailing address of corresponding author	X		1-12
Contributions	3b	Describe contributions of protocol authors and identify the guarantor of the review	X		556-558
**Amendments**	4	If the protocol represents an amendment of a previously completed or published protocol, identify as such and list changes; otherwise, state plan for documenting important protocol amendments	X		549-551
**Support**					
Sources	5a	Indicate sources of financial or other support for the review	X		560-565
Sponsor	5b	Provide name for the review funder and/or sponsor	X		560-565
Role of sponsor/funder	5c	Describe roles of funder(s), sponsor(s), and/or institution(s), if any, in developing the protocol	X		568-571
**INTRODUCTION**					
**Rationale**	6	Describe the rationale for the review in the context of what is already known	X		61-178
**Objectives**	7	Provide an explicit statement of the question(s) the review will address with reference to participants, interventions, comparators, and outcomes (PICO)	X		180-195
**METHODS**					

For peer review only

BioMed Central The Open Access Publisher

2

Section/topic	#	Checklist item	Information reported Yes	No	Line number(s)
**Eligibility criteria**	8	Specify the study characteristics (e.g., PICO, study design, setting, time frame) and report characteristics (e.g., years considered, language, publication status) to be used as criteria for eligibility for the review	X	☐	198-302; 342-395
**Information sources**	9	Describe all intended information sources (e.g., electronic databases, contact with study authors, trial registers, or other grey literature sources) with planned dates of coverage	X	☐	310-319
**Search strategy**	10	Present draft of search strategy to be used for at least one electronic database, including planned limits, such that it could be repeated	☐	X	
STUDY RECORDS					
Data management	11a	Describe the mechanism(s) that will be used to manage records and data throughout the review	X	☐	344-348
Selection process	11b	State the process that will be used for selecting studies (e.g., two independent reviewers) through each phase of the review (i.e., screening, eligibility, and inclusion in meta-analysis)	X	☐	330-340
Data collection process	11c	Describe planned method of extracting data from reports (e.g., piloting forms, done independently, in duplicate), any processes for obtaining and confirming data from investigators	X	☐	344-349
**Data items**	12	List and define all variables for which data will be sought (e.g., PICO items, funding sources), any pre-planned data assumptions and simplifications	X	☐	350-395
**Outcomes and prioritization**	13	List and define all outcomes for which data will be sought, including prioritization of main and additional outcomes, with rationale	X	☐	234-302
**Risk of bias in individual studies**	14	Describe anticipated methods for assessing risk of bias of individual studies, including whether this will be done at the outcome or study level, or both; state how this information will be used in data synthesis	X	☐	426-442
DATA					
**Synthesis**	15a	Describe criteria under which study data will be quantitatively synthesized	X	☐	446-452
	15b	If data are appropriate for quantitative synthesis, describe planned summary measures, methods of handling data, and methods of combining data from studies, including any planned exploration of consistency (e.g., I^2, Kendall's tau)	X	☐	482-491
	15c	Describe any proposed additional analyses (e.g., sensitivity or subgroup analyses, meta-regression)	X	☐	523-541
	15d	If quantitative synthesis is not appropriate, describe the type of summary planned	☐	X	
**Meta-bias(es)**	16	Specify any planned assessment of meta-bias(es) (e.g., publication bias across studies, selective reporting within studies)	X	☐	493-497
**Confidence in cumulative evidence**	17	Describe how the strength of the body of evidence will be assessed (e.g., GRADE)	X	☐	506-520

For peer review only

BioMed Central
The Open Access Publisher

BMJ Open

Vector control for malaria prevention during humanitarian emergencies: protocol for a systematic review and meta-analysis

Journal:	*BMJ Open*
Manuscript ID	bmjopen-2020-046325.R1
Article Type:	Protocol
Date Submitted by the Author:	09-Jun-2021
Complete List of Authors:	Messenger, Louisa; London School of Hygiene & Tropical Medicine, Disease Control<br>Furnival-Adams, Joanna; London School of Hygiene & Tropical Medicine, Disease Control<br>Pelloquin, Bethanie; London School of Hygiene & Tropical Medicine, Disease Control<br>Rowland, Mark; London School of Hygiene & Tropical Medicine, Disease Control
**Primary Subject Heading**:	Global health
Secondary Subject Heading:	Public health
Keywords:	Public health < INFECTIOUS DISEASES, ACCIDENT & EMERGENCY MEDICINE, Epidemiology < INFECTIOUS DISEASES, Tropical medicine < INFECTIOUS DISEASES

SCHOLARONE™
Manuscripts

I, the Submitting Author has the right to grant and does grant on behalf of all authors of the Work (as defined in the below author licence), an exclusive licence and/or a non-exclusive licence for contributions from authors who are: i) UK Crown employees; ii) where BMJ has agreed a CC-BY licence shall apply, and/or iii) in accordance with the terms applicable for US Federal Government officers or employees acting as part of their official duties; on a worldwide, perpetual, irrevocable, royalty-free basis to BMJ Publishing Group Ltd ("BMJ") its licensees and where the relevant Journal is co-owned by BMJ to the co-owners of the Journal, to publish the Work in this journal and any other BMJ products and to exploit all rights, as set out in our licence.

The Submitting Author accepts and understands that any supply made under these terms is made by BMJ to the Submitting Author unless you are acting as an employee on behalf of your employer or a postgraduate student of an affiliated institution which is paying any applicable article publishing charge ("APC") for Open Access articles. Where the Submitting Author wishes to make the Work available on an Open Access basis (and intends to pay the relevant APC), the terms of reuse of such Open Access shall be governed by a Creative Commons licence – details of these licences and which Creative Commons licence will apply to this Work are set out in our licence referred to above.

Other than as permitted in any relevant BMJ Author's Self Archiving Policies, I confirm this Work has not been accepted for publication elsewhere, is not being considered for publication elsewhere and does not duplicate material already published. I confirm all authors consent to publication of this Work and authorise the granting of this licence.

For peer review only

Vector control for malaria prevention during humanitarian emergencies: protocol for a systematic review and meta-analysis

Louisa A. Messenger[1], Joanna Furnival-Adams[1,2], Bethanie Pelloquin[1,3], Mark Rowland[1]

[1] Department of Disease Control, Faculty of Infectious Tropical Diseases, London School of Hygiene and Tropical Medicine, London, United Kingdom
[2] ISGlobal, Hospital Clinic – Universitat de Barcelona, Rosello 132, 08036, Spain
[3] School of Tropical Medicine and Global Health, University of Nagasaki, Nagasaki, Japan

Contact address: Louisa A. Messenger, Department of Disease Control, Faculty of Infectious Tropical Diseases, London School of Hygiene and Tropical Medicine, London, United Kingdom.
Louisa.messenger@lshtm.ac.uk

Abstract (300 words)

Introduction Humanitarian emergencies, of either natural or anthropogenic origins, are equivalent to major disasters, which can lead to population displacement, food insecurity and health system disruptions. Almost two-thirds of persons affected by humanitarian emergencies inhabit malaria endemic regions, particularly the World Health Organization (WHO) African Region, which currently accounts for 93% and 94% of malaria cases and deaths, respectively. As of late 2020, the United Nations Refugee Agency estimates there are globally 79.5 million forcibly displaced people, including 45.7 million internally displaced people, 26 million refugees, 4.2 million asylum-seekers and 3.6 million Venezuelans displaced abroad.

Methods and analyses A systematic review and meta-analysis will be conducted to evaluate the impact of different vector control interventions on malaria disease burden during humanitarian emergencies. Published and grey literature will be systematically retrieved from ten electronic databases and three clinical trials registries. A systematic approach to screening, reviewing and data extraction will be applied based on the Preferred Reporting Items for Systematic Reviews and Meta-Analysis (PRISMA) guidelines. Two review authors will independently assess full-text copies of potentially relevant articles based on inclusion criteria. Included studies will be assessed for risk of bias according to Cochrane and certainty of evidence using the GRADE approach. Eligible studies with reported or measurable risk ratios or odds ratios with 95% confidence intervals will be included in a meta-analysis. Sub-group analyses, including per study design, emergency phase, and primary mode of intervention, may be performed if substantial heterogeneity is encountered.

Ethics and dissemination Ethical approval is not required by the London School of Hygiene and Tropical Medicine to perform secondary analyses of existing anonymous data. Study findings will be disseminated via open-access publications in peer-reviewed journals, presentations to stakeholders and international policy makers, and will contribute to the latest WHO guidelines for malaria control during humanitarian emergencies.

PROSPERO registration number CRD42020214961.

For peer review only - http://bmjopen.bmj.com/site/about/guidelines.xhtml

Strengths and limitations of this study

- ⇒ This will be the first systematic review and meta-analysis of the impact of different vector control interventions on malaria disease burden during humanitarian emergencies, providing a comprehensive global evidence summary that can be updated as new evidence emerges.
- ⇒ Inclusion of a broad range of study types will allow the identification of studies from a diverse range of humanitarian contexts.
- ⇒ By definition, humanitarian emergencies are unpredictable and volatile and consequently these settings can be restrictive with respect to study design, limiting the number of community-level randomised control trials with epidemiological outcomes which can be considered in this review.
- ⇒ Lack of a sensitive literature search strategy may result in a large number of titles and abstracts to screen initially.

INTRODUCTION

Malaria in humanitarian emergencies

Malaria is a life-threatening parasitic disease caused by *Plasmodium* species and transmitted by female *Anopheles* mosquitoes.[1] In 2018, there were an estimated 228 million cases of malaria worldwide, including 405,000 deaths.[1]. As defined by the WHO, a humanitarian emergency is a calamitous situation in which the functioning of a community or society is severely disrupted, causing human suffering and material loss that exceeds the affected population's ability to cope using its own resources.[2] Both natural (e.g. earthquakes, floods or tsunamis) and anthropogenic (e.g. violent political conflicts) hazards can lead to humanitarian emergencies, which may involve population displacement, food insecurity and disruptions to health systems; all of these factors contribute to excess mortality and morbidity among affected communities. While natural disasters may be short-lived, complex emergencies are often protracted, characterized by having complex social, political and economic origins, breakdown of governmental authority and infrastructures and often armed political conflict and human rights abuses.[2] Globally, the United Nations Refugee Agency (UNHCR) currently estimates there are 79.5 million forcibly displaced people[3]; with almost two-thirds of persons affected by humanitarian emergencies inhabiting malaria endemic regions.[4] (Figure 1).

Figure 1. Left: Global map of internally displaced people (green) and refugee (red) populations, 2020. The UNHCR currently estimates there are 79.5 million forcibly displaced people, including 45.7 million internally displaced people, 26 million refugees, 4.2 million asylum-seekers and 3.6 million Venezuelans displaced abroad.[3] **Right:** Global map of *Plasmodium falciparum* parasite rate in children aged 2-10 years old, 2000-2017.[5]

Mass population displacement can increase the risk of epidemics and incidence of severe malaria, especially when immunologically-naive individuals with little to no prior malaria exposure move to areas of more intense transmission. These populations are rendered vulnerable to epidemic malaria by their lack of protective immunity, increased concentration of people in exposed settings, inadequate health services, breakdown of national malaria control programmes, loss or emigration of skilled healthcare workers, insufficient access to effective treatment and control measures, food scarcity, and concomitant infections,

For peer review only - http://bmjopen.bmj.com/site/about/guidelines.xhtml

malnutrition and anaemia.[2] During humanitarian emergencies, malaria morbidity and mortality can contribute to the breakdown of health services and local case management, which can both worsen and be a source of the emergency.

Humanitarian emergency features and settings

Humanitarian emergencies can be classified by their speed of onset, duration and setting, which all have implications for infectious disease control strategies (Figure 2). Rapid- and slow-onset emergencies describe a sudden or gradual deteriorating situation, respectively, while protracted (chronic) emergencies involve consistently high levels of humanitarian need. The criteria of the acute phase (Phase 1 and 2 in Figure 2), are elevated mortality rates, poor access to effective healthcare for the affected population, response needs that are beyond local or national capacity, and possible breakdown of normal coordination and response mechanisms. The post-acute phase (Phase 3), includes a reduction in peak mortality rates and improved access to humanitarian aid and healthcare. The recovery phase (Phase 4), is characterised by resumption of government, the withdrawal of humanitarian agencies, and introduction of development UN and NGO programmes and agencies. However, returning refugees may also re-integrate, bringing malaria with them, and depending on the level of strategic interest this phase may or may not be neglected internationally. Protracted emergencies can remain entrenched in the post-acute/early-recovery phases or cycle between acute and post-acute phases indefinitely.

Figure 2. Phases of sudden-onset, slow-onset or protracted (chronic) humanitarian emergencies. Time is indicated in days (D), weeks (W) and months (M). Adapted from [2].

Malaria vector control during humanitarian emergencies

During the acute phase of a humanitarian emergency, the first priorities for malaria control are effective case management, with prompt diagnosis and treatment.[2] Choice of vector control intervention will be determined by a number of key factors including, malaria infection risk and transmission dynamics, population demographics, prior malaria exposure, behaviour and mobility, vector species composition, vector feeding and resting preferences and emergency settings and infrastructure (Table 1).

WHO recommended malaria control interventions, insecticide-treated nets (ITNs) and indoor residual spraying (IRS), are usually the first line preventative measures considered. Effective case management can be supplemented with long-lasting insecticidal net (LLIN) distributions, first targeting the most vulnerable populations (e.g. pregnant women and children under-5 years old), with the goal of achieving and maintaining universal community-level coverage.[6-20] IRS can be applied in well-organised, politically-stable settings, such as transit camps, but is generally not feasible when dwellings are scattered widely, of a temporary nature, or constructed with surfaces that are unsuitable for spraying.[21] IRS may be more appropriate for protecting larger refugee populations in more compact and stable settings where housing is more permanent and structurally sound, and there is an absence of alternative resting sites.[22-24] Targeted IRS can also supplement LLINs and be used together for insecticide resistance management.[25 26]

A suite of other community-level and personal vector control interventions has been designed for deployment during humanitarian emergencies, which will be considered in this review. One strategy has been to exploit utilitarian shelter materials, often provided in the early stages of emergencies, such as plastic sheeting, tarpaulins and tents, as mechanisms of insecticide delivery.[27-30] Additional opportunities identified to apply insecticide among at-

For peer review only - http://bmjopen.bmj.com/site/about/guidelines.xhtml

risk populations have included distributing insecticide treated bed sheets and blankets, clothing, hammocks and wall lining,[31-39] and use of topical insecticides for human populations [40-42] or to treat community livestock.[43]

Most interventions act by delivering a lethal dose of insecticide to the adult mosquito, protecting the host from exposure to a potentially infective bite. Some of these interventions, notably LLINs and IRS, may also have a community wide benefit (in areas of vector susceptibility to the active ingredients), decreasing mosquito density and longevity, and ultimately interrupting malaria transmission. The insecticide used is dependent on the intervention and resistance profiles of local vector species.[44 45] Other interventions, such as topical repellents, work by preventing mosquitoes from detecting the host, by disrupting olfactory reception.[46] These interventions only provide individual protection and do not impact vectorial capacity.[44]

There are multiple factors involved in ensuring a successful vector control programme during humanitarian emergencies. The intervention(s) selected must be feasible and consider local vector species, human resources, funding, logistics and population mobility.[2] Other benefits of vector control interventions, which may make them more acceptable to the user, include reducing flies in households and nuisance biting.[47] and improved health and yields of treated livestock.[43] Some interventions may have low acceptability, for example, sleeping under an ITN or treated blanket/bedsheet in hot, humid conditions, some types of treated clothing may be culturally inappropriate, topical repellents may cause irritation to individuals, and there may be a decrease in adherence over time to interventions which require sustained behavioural changes.[41]

Why it is important to do this review

To date, there has been insufficient, systematic syntheses of evidence for the WHO to make recommendations for deployment of malaria vector control interventions in humanitarian settings. While a number of aforementioned interventions have either been evaluated during or developed specifically for use in humanitarian emergencies, the often unpredictable and volatile nature of these settings can often be restrictive in terms of designing experimental studies. Given these logistical constraints, a range of both observational and experimental study designs will be considered.

Objectives

This study aims to evaluate the impact of different vector control interventions on malaria disease burden during humanitarian emergencies.

Primary objective is:

- To evaluate the impact of different vector control interventions on malaria disease burden during humanitarian emergencies.

Secondary objectives are:

- To explore whether the impact of the different vector control interventions varies with the phase of humanitarian emergency (acute/post-acute, protracted and recovery/post-conflict phases).

For peer review only - http://bmjopen.bmj.com/site/about/guidelines.xhtml

- To explore whether the impact of the different vector control interventions varies with the level of malaria transmission (low, <50% parasite rate in children; and high, >50% parasite rate in children).
- To explore whether the impact of the different vector control interventions varies with the level of coverage during a humanitarian emergency.

METHODS AND ANALYSES

The following types of studies for inclusion in this review:

- Cluster-randomised controlled trials (c-RCTs).
- Cluster-randomised cross-over studies.
- Cluster-randomised studies using a stepped-wedge design.
- Controlled before-and-after studies (CBAs).
- Cohort studies (prospective or retrospective).
- Non-randomised cross-over studies.
- Programmatic evaluations.
- Cross-sectional studies.
- Case-control studies.
- Case series.
- Interrupted time series (ITS).

Experimental hut trials, other phase II studies or user acceptability surveys (including, quantitative knowledge, attitude and practices (KAP) questionnaires or qualitative observations, in-depth interviews (IDIs) and focus group discussions (FGDs)) will be summarised descriptively.

Type of participants

Individuals of all age groups affected by humanitarian emergencies in malaria endemic regions; including those that have been internally displaced by humanitarian emergencies as well as host communities. A humanitarian emergency is defined as a calamitous situation, of either natural (including famine, flooding (localised or widespread), storms, cyclones, earthquake, tsunami) or anthropogenic origin (may include widespread (i.e. affecting whole regions/countries) or geographically confined conflict (i.e. affecting only parts of a country/or multiple countries, e.g. the West African Islamic terrorist crisis affecting border regions of Mali, Niger, Nigeria, Burkina Faso, Cameroon and Chad), or persecution of a specific ethic group (e.g. Bosnian, Rohingya and Rwanda genocides), in which the functioning of a community or society (whether a subsection or whole population) was severely disrupted, causing human suffering and material loss that exceeded the affected population's ability to cope using its own resources.[2] Studies with military personnel or peacekeepers, in demilitarized zones, with migrant, tribal or nomadic populations or temporary/seasonal labourers (e.g. forest or dam workers, gold or gem miners) will be excluded.

Type of interventions

Any malaria-specific vector control intervention, which has been evaluated during a humanitarian emergency, will be considered for inclusion (Table 2).

Type of outcome measures

For peer review only - http://bmjopen.bmj.com/site/about/guidelines.xhtml

To be eligible for inclusion, studies must report at least one of the following primary outcomes.

Primary outcomes

- Malaria case incidence: measured as number of cases per unit of time or the number of new uncomplicated malaria cases. We will use site-specific definitions as long as they have demonstrated (i) a fever or history of fever, and (ii) confirmed parasitaemia (by blood smear microscopy, rapid diagnostic test (RDT), or polymerase chain reaction (PCR)).
- Malaria infection incidence: measured as count per person per unit of time or the number of new infections (individuals must have confirmed parasitaemia by blood smear, RDT or PCR).
- Parasite prevalence (clinical and subclinical malaria): the proportion of surveyed individuals with confirmed parasitaemia at the community-level.

Secondary outcomes

Epidemiological

- All-case mortality.
- Severe malaria, based on confirmed malaria diagnosis (individuals must have confirmed parasitaemia by blood smear, RDT or PCR) plus the presence of clinical or laboratory features of vital organ dysfunction (these include impaired consciousness, coma, convulsions, respiratory distress, shock (systolic blood pressure <70 mmHg in adults, <50 mmHg in children), jaundice, haemoglobinuria, hypoglycaemia, severe metabolic acidosis and anaemia).
- Anaemia prevalence as per WHO cut-offs, based on haemoglobin measurements taken in community surveys.

Entomological

- Entomological inoculation rate (EIR): the estimated number of bites by infectious mosquitoes per person per unit of time. This is calculated using the human biting rate (the number of mosquitoes biting an individual over a stated period, measured directly using human baits or indirectly using light traps, knock-down catches, entry or exit traps, baited huts, or other methods of biting rate determination) multiplied by the sporozoite rate.
- Adult mosquito density: measured by a technique previously shown to be appropriate for the particular vector species (e.g. human baits, light-traps, knock-down catches, entry or exit traps, baited huts, or other methods).
- Sporozoite rate: measured as the number of caught adult mosquitoes positive for malaria sporozoites. Sporozoites can be detected through either molecular or immunological methods.

Operational

- Intervention durability: measured by a technique previously shown to be appropriate, (e.g. a time-series of WHO cone bioassays to monitor IRS residual activity or ITN/LLIN bioefficacy plus HPLC to determine intervention insecticidal content).

For peer review only - http://bmjopen.bmj.com/site/about/guidelines.xhtml

Adverse events

- Any indicators of adverse effects of the intervention, including the following:
  - Reports of toxicity to humans/animals
  - Environmental impacts, such as changes to the biodiversity and ecosystem due to insecticidal use
  - Changes to the levels of phenotypic/molecular insecticide resistance, assessed using standard WHO cylinder assays and/or Centers for Disease Control and Prevention (CDC) bottle bioassays/molecular techniques
  - Changes in mosquito species composition, e.g. species replacement or behaviour that reduces the efficacy of vector control interventions, e.g. an increase in exophily, exophagy, zoophily, or changes in biting time

Unintended effects

- Impact on human behaviour, such as changes to individuals' sleeping arrangements or use of other vector control interventions.
- Other vector-borne disease case incidence (for example, dengue and leishmaniasis).

Search methods for identification of studies

We will attempt to identify all relevant trials and studies, regardless of language, date or publication status (published, unpublished, in press and in progress).

Electronic searches

We will search the following databases: Cochrane Infectious Disease Group Specialized Register; Central Register of Controlled Trials (CENTRAL), published in the Cochrane Library; MEDLINE (OvidSP); Africa-Wide Information (EBSCO); the WHO Global Index Medicus; the Science Citation Index Expanded (Web of Science); the Conference Proceedings Citation Index – Science (Web of Science); Embase (OvidSP); Global Health (CABI); and LILACS. We will also search the WHO International Clinical Trials Registry Platform (www.who.int/ictrp/search/en), ClinicalTrials.gov (www.clinicaltrials.gov), and the ISRCTN registry (www.isrctn.com) to identify ongoing trials.

Searching other resources

We will contact past and present personnel at key stakeholders, donors, global policy makers, technical groups, non-governmental organisations (NGOs), manufacturers, implementers and researchers in the field to identify sources of unpublished data. We will also check the reference lists of studies identified by the above methods.

Data collection and analysis

Selection of studies

Study selection will follow PRISMA guidelines. Two review authors will independently assess the titles and abstracts of studies identified by the literature searches. These two review authors will assess full-text copies of potentially relevant articles based on inclusion criteria. Studies will be eligible for inclusion in the systematic review and meta-analysis if they satisfy all criteria: the study population consisted of individuals of either all age groups, or children of specified age groups (i.e. <5 years, >5-15 years), affected by humanitarian emergencies

For peer review only

(any phase) in malaria endemic regions; any malaria-specific vector control intervention was evaluated; and the primary outcome of interest was malaria infection risk, measured as case incidence, infection incidence or parasite prevalence. Studies will be eligible for inclusion in secondary analyses, where none of the primary malaria risk indicators were measured, but at least one of the following secondary outcomes were reported: all-cause mortality, severe malaria, anaemia prevalence, entomological inoculation rate, adult mosquito density, sporozoite rate, intervention durability, occurrence of adverse events and user acceptability/usage.

We will compare the results of our assessments and resolve any disagreements by discussion and consensus, with arbitration by a third review author, if necessary. We will ensure that multiple publications of the same study are included once. We will list excluded studies, together with their reasons for exclusion, in a 'Characteristics of excluded studies' table; any highly relevant studies which did not meet the inclusion criteria, may be included in a separate annex. The results of the study selection process will be illustrated in a PRISMA diagram.

Data extraction and management

Two review authors will independently extract information from the included studies into a pre-piloted database, in which study settings, methods and results from identified studies will be recorded. Disagreements in data extraction will be resolved by discussion and consensus between the two review authors and arbitration by a third review author, if necessary. In case of missing data, we will contact the original study author(s) for clarification. Data will be extracted on the following:

- Lead author and publication year.
- Study period.
- Study design: type of study; method of participant selection (for c-RCTs: adjustment for clustering); cluster size; number of clusters; cross over; sample size.
- Study participants: population size; origins and length of time in setting (with respect to the particular emergency situation); characteristics: age, sex, ethnicity, recruitment rates, withdrawal, loss to follow up, socioeconomic status; levels of prior malaria exposure; proportion of vulnerable individuals; prevalence of co-morbidities.
- Humanitarian emergency context: geographic country or area affected; underlying cause(s) of emergency and relevant mitigating factors; structure and layout of residence area (e.g. formal camp, informal settlement, open/closed); type of accommodation or housing available in study area; displacement dynamics and mobility; road access and condition; details of the political and logistical constraints encountered during different phases of humanitarian emergencies and their impact on the feasibility and choice of vector control intervention(s).
- Epidemiological context: malaria transmission intensity; incidence; prevalence; *Plasmodium* species.
- Intervention: description of intervention (active ingredient, dose, formulation, intervention format, frequency and timing of implementation); coverage, usage and adherence; any reports of refusals and reasons why.
- Cointerventions: details of any other malaria or vector-specific control interventions (such as, mass drug administration, active case detection, health education, untreated bednets); coverage and adherence; any reports of refusals and reasons why.
- Delivery mechanism(s): route of intervention deployment (for example, community-wide dissemination, health facility-based distributions, integration with other essential services); any additional information related to the method of implementation,

For peer review only

including who implemented the intervention and who was targeted (the general population or a sub-population).

- Outcome measurements: definition of outcome; diagnostic or surveillance method; passive or active case detection; duration of follow-up; time points at which outcomes were assessed; number of events; number of participants or unit time; statistical power; unit of analysis; incomplete outcomes/missing data.
- Other: primary and secondary vector(s) species; vector(s) behaviour (population seasonality, adult habitat, peaking biting times, exophilic/endophilic, exophagic/endophagic, anthropophilic/zoophilic); method of vector collection(s); baseline measures of phenotypic insecticide resistance and any measures during the study period; baseline measures of genotypic insecticide resistance and any measure during the study period; any measure of vector sporozoite rate; identification of vector bloodmeal(s); vector parity and age structure; any measure of user acceptability collected during the trial and reported by the treatment arm, including cross-sectional survey data of reported acceptability and qualitative data on opinions about the intervention.
- Identification of major limitations in design, analysis, results, and any aspects that would limit generalizability of results.
- Source of funding of the study; reported conflicts of interest of authors.

For dichotomous outcomes, we will extract the number of participants experiencing each outcome and number of participants in each study group. For continuous outcomes, we will extract number of participants in each study group, the mean and a measure of variance (for example, standard deviation or standard error). For count/rate data outcomes, we will extract the number of outcomes in the treatment and control groups, and the total person time at risk in each group (or the rate ratio), and a measure of variance. For c-RCTs, we will record the number of clusters randomised, number of clusters analysed, measure of effect e.g. risk ratio, odds ratio or mean difference, with confidence intervals (CIs) or standard deviations, number of participants and the intra-cluster correlation coefficient (ICC) value. For non-randomised studies, we will extract adjusted measures of effects that attempt to control for confounding. If there are no adjusted measures reported, then we will extract unadjusted measures. We will group studies for analyses according to the intervention under evaluation.

Assessment of risk of bias in included studies

Two review authors will independently assess the risk of bias for each study, consulting a third review author if necessary. For c-RCTs, we will use the Cochrane ‘Risk of bias’ tool, as well as the six additional criteria listed in Section 23.1.2 of the *Cochrane Handbook for Systematic Reviews of Interventions* that relate specifically to c-RCTs.[48] For randomised cross-over trials, we will use the Cochrane 'Risk of bias' tool, as well as the additional criteria listed in Section 23.2.3 of the *Cochrane Handbook for Systematic Reviews of Interventions* that relate specifically to randomised cross-over trials. For non-randomised controlled studies, we will use the Cochrane Effective Practice and Organisation of Care (EPOC) 'Risk of bias' tool. Biases will be classified as ‘low’, ‘high’ or ‘unclear’ and summarised in graphs. For observational studies, we will use the Newcastle-Ottawa Scale (NOS).[49] We will assess risk of bias through a hierarchy of domains, starting with ‘critical’, then ‘serious’, ‘moderate’, and ‘low’. If any domain reaches critical risk of bias we will not continue with the assessment. We will make a risk of bias assessment for each outcome. If risk of bias is critical it will be excluded from the review. Highly relevant studies with a critical risk of bias may be included in a separate annex.

For peer review only - http://bmjopen.bmj.com/site/about/guidelines.xhtml

Measures of treatment effect

We will compare intervention and control data using (preferably) risk ratios or odds ratios if the outcome is dichotomous. For continuous data, we will present the mean difference (when studies use the same outcome measurement scale, and SMD when studies use different outcome measurement scale); and for count/rate data, we will use rate ratios. We will use adjusted measures of effect to summarise treatment effects from non-randomised studies. We will present all results with their associated 95% CIs. We will report any accounts that signal adverse effects.

Unit of analysis issues

For c-RCTs, or non-randomised cluster trials, we will extract adjusted measures of effect where possible. If the study authors did not perform any adjustment for clustering, we will adjust the raw data ourselves using an ICC value. If an ICC is not reported, we will estimate this, with reference to similar studies, if possible. We will not present results from c-RCTs that are not adjusted for clustering. If we estimate the ICC, we will perform sensitivity analyses to investigate the robustness of our analyses. If we identify studies for inclusion that have multiple intervention arms, we will include data from these studies by either combining treatment arms, or by splitting the control group so that we only include these participants in the meta-analysis once. We will consider the level at which randomization occurred, such as cross-over trials, c-RCTs, and multiple observations for the same outcome. For randomised cross-over trials, where neither carry-over nor period effects are identified as problematic, we will use a paired t-test for the analysis of continuous data from two-period, two-armed cross-over trials.

Dealing with missing data

In case of missing data, we will apply available-case analysis and will include data on known results, as well as 'non-adherent' participants, excluded from the primary study authors' analysis, but for whom data are available.[50] 'Non-adherent' participant data may be included in "intention-to-treat" or "as-treated" analyses, as appropriate.[50] The denominator will vary according to the total number of participants who had data recorded for the specific outcome as well as the total number of excluded participants included per analysis type. For outcomes with no missing data, we plan to perform analyses on an "intention-to-treat" basis. We will include all participants randomised to each group in the analyses and will analyse participants in the group to which they were randomised. If further data are needed, respective authors, companies or organisations will be contacted to obtain missing data.

Assessment of heterogeneity

Heterogeneity will be assessed by visually examining the overlap of study CIs, applying Chi^2 and I^2 statistics. A Chi^2 test result with P value < 0.1 will indicate significant heterogeneity. Interpretation of I^2 will follow the classification: 30% to 60%: may represent moderate heterogeneity; 50% to 90%: substantial heterogeneity; 75% to 100%: considerable heterogeneity. If we observe substantial heterogeneity we will perform further subgroup analysis. If appropriate we will explore clinical and methodological heterogeneity through consideration of the trial populations, methods, and interventions and by visualization of trial results.

Assessment of reporting biases

For peer review only - http://bmjopen.bmj.com/site/about/guidelines.xhtml

We will construct a funnel plot to assess reporting biases when performing an analysis of 10 or more studies. We will explore reasons for funnel plot asymmetry, including true heterogeneity of the effect with respect to trial size, poor methodological design (and hence bias of small trials), and publication bias, and will interpret results accordingly.

Data synthesis

We will analyse data using Review Manager 5.[51] We will use fixed-effect meta-analysis to combine data if heterogeneity is absent. If considerable heterogeneity is present, we will combine data using random-effects meta-analysis and report an average treatment effect. We will decide whether to use fixed-effect or random-effects models based on the consideration of clinical and methodological heterogeneity between trials.

Certainty of the evidence

We will assess the certainty of evidence using the GRADE approach.[52] Evidence will start as high-certainty. However, if there is bias due to confounding, missing data or selection of the report result, outcomes will be marked as moderate, high or unclear risk of bias and the evidence will be rated down accordingly to low-certainty evidence.

For c-RCTs, we will rate each important outcome as described by.[53] c-RCTs start as high quality evidence but can be downgraded if there are valid reasons within the following five categories: risk of bias, imprecision, inconsistency, indirectness, and publication bias. Non-randomized studies can also be upgraded if there is a large effect, and if all plausible residual confounding would reduce a demonstrated effect or would suggest a spurious effect if no effect was observed.[53] We will summarise our findings in a 'Summary of findings' table.

We will summarise qualitative findings on consumer views e.g. user acceptability narratively. If there are a sufficient number of included studies, two review authors will independently code the studies, and use thematic synthesis to identify themes and subthemes.

Subgroup analysis and investigation of heterogeneity

We will stratify the analysis by study design, phase of humanitarian emergency (acute/post-acute, protracted and recovery/post-conflict phases) and primary mode of intervention action. Where substantial heterogeneity exists, we plan to perform the following subgroup analysis:

- Intervention coverage (appropriate levels to be determined with respect to the primary mode of action of each intervention, e.g. community-wide mass effect *vs.* personal protection).
- Participants (<5 years, pregnant women, adult, mixed age groups).
- Geographical region.
- Malaria vector species behaviour.
- Malaria transmission dynamics/seasonality.

Sensitivity analysis

We will perform sensitivity analysis on the primary outcome, to observe the effect of excluding trials at high risk of bias (for incomplete outcome data). If the ICC value is

For peer review only - http://bmjopen.bmj.com/site/about/guidelines.xhtml

estimated, we will undertake sensitivity analyses to investigate the impact of varying the ICC value on meta-analysis results.

PATIENT AND PUBLIC INVOLVEMENT

No patients were involved in the design of this study.

ETHICS AND DISSEMINATION

This systematic review and meta-analysis will consist of secondary analyses of existing anonymous data and meets the criteria for waiver of ethics review by the London School of Hygiene and Tropical Medicine. As this research is based on previously published data, there will be no patient and public involvement in the design, interpretation or dissemination of study findings. This study protocol was drafted according to the Preferred Reporting Items for Systematic Reviews and Meta-Analyses Protocols (PRISMA-P) checklist.[54] Any amendments to this protocol will be documented and published alongside the results of the systematic review and meta-analysis. Study findings will be disseminated via open-access publications in peer-reviewed journals, presentations to stakeholders and relevant national and international policy makers and will contribute to the latest WHO guidelines for malaria control during humanitarian emergencies.

Authors' contributions

LAM, JFA, BP and MR wrote the full text of the protocol. All review authors read and approved the final protocol draft.

Funding statement

This work was supported by the World Health Organization Global Malaria Programme (WHO-GMP). LAM, JFA and MR are supported by the DFID/MRC/NIHR/Wellcome Trust Joint Global Health Trials Scheme. LAM and MR are also supported by UNITAID/Global Fund and DFID-RPC. BP is supported by the LSHTM-Nagasaki University Joint PhD Programme for Global Health. We are very grateful to Jane Falconer, Information Specialist, at the London School of Hygiene and Tropical Medicine, for help with the literature search strategy, and to Drs. Jan Kolaczinski, Jennifer Stevenson and Elie Akl (WHO-GMP) and Drs. Natacha Protopopoff and Natasha Howard (LSHTM) for valuable protocol suggestions. The funder of the study had no role in study design, data collection, data analysis, data interpretation, or writing of the report. The corresponding author had full access to all the data in the study and had final responsibility for the decision to submit for publication.

Competing interests statement.

The authors declare no competing interests.

References

1. World Health Organization 2019. World Malaria Report. Geneva, Switzerland: World Health Organization 2019.
2. World Health Organization 2013. Malaria control in humanitarian emergencies: an inter-agency field handbook. Second edition Geneva: 2013.

For peer review only - http://bmjopen.bmj.com/site/about/guidelines.xhtml

3. The UN refugee Agency (UNHCR), 2020. Figures at a glance. Accessible from: https://www.unhcr.org/uk/figures-at-a-glance.html Accessed September 5th 2020.
4. Anderson J, Doocy S, Haskew C, *et al.* The burden of malaria in post-emergency refugee sites: a retrospective study. *Confl Health* 2011;5:17.
5. The Malaria Atlas Project (MAP), 2020. Interactive map. Accessible from: https://malariaatlas.org/ Accessed September 5th 2020.
6. Spencer S, Grant AD, Piola P, *et al.* Malaria in camps for internally-displaced persons in Uganda: evaluation of an insecticide-treated bednet distribution programme. *Trans R Soc Trop Med Hyg* 2004;98(12):719-27.
7. Richards AK, Banek K, Mullany LC, *et al.* Cross-border malaria control for internally displaced persons: observational results from a pilot programme in Eastern Burma/Myanmar. *Trop Med Int* Health 2009;14(5):512-21.
8. Smithuis FM, Kyaw MK, Phe UO, *et al.* The effect of insecticide-treated bed nets on the incidence and prevalence of malaria in children in an area of unstable seasonal transmission in western Myanmar. *Malaria J* 2013;12:363.
9. Dolan G, ter Kuile FO, Jacoutot V, White NJ, Luxemburger C, Malankirii L, Chongsuphajaisiddhi T, Nosten F. Bed nets for the prevention of malaria and anaemia in pregnancy. *Trans R Soc Trop Med Hyg* 1993;87:620-26.
10. Luxemburger C, Perea WA, Delmas G, Pruja C, Pecoul B, Moren A. Permethrin-impregnated bed nets for the prevention of malaria in schoolchildren on the Thai-Burmease border. *Trans R Soc Trop Med Hyg* 1994;88:155-59.
11. Rowland M, Bouma M, Ducornez D, Durrani N, Rozendaal J, Schapira A, Sondorp E. Pyrethroid-impregnated bed nets for personal protection against malaria for Afghan refugees. *Trans R Soc Trop Med Hyg* 1996;90:357-61.
12. Brooks HM, Jean Paul MK, Claude KM, Mocanu V, Hawkes MT. Use and disuse of malaria bed nets in an internally displaced persons camp in the Democratic Republic of the Congo: a mixed methods study. *PLoS One* 2017; 12(9):e0185290.
13. Brooks HM, Jean Paul MK, Claude KM, Houston S, Hawkes MT. Malaria in an internally displaced persons camp in the Democratic Republic of the Congo. *Clin Infect Dis* 2017;65(3):529-30.
14. Chan CW, Iata H, Yaviong J, Kalkoa M, Yamar S, Taleo G, Isozumi R, Fukui M, Aoyama F, Pomer A, Dancause KN, Kaneko A. Surveillance for malaria outbreak on malaria-eliminating islands in Tafea Province, Vanuatu after Tropical Cyclone Pam in 2015. *Epidemiol Infect* 2017;145:41-5.
15. Protopopoff N, Van Herp M, Maes P, Reid T, Baza D, D'Alessandro U, Van Bortel W, Cossemans M. Vector control in a malaria epidemic occurring within a complex emergency situation in Burundi: a case study. *Malar J* 2007;6:93.
16. Ma C, Claude MK, Kibendelwa ZT, Brooks H, Zheng X, Hawkes M. Is maternal education a social vaccine for childhood malaria infection? A cross-sectional study from war-torn Democratic Republic of Congo. *Pathog Glob Health* 2017;111(2):98-106.
17. Charchuk R, Jean Paul MK, Claude KM, Houston S, Hawkes MT. Burden of malaria is higher among children in an internal displacement camp compared to a neighbouring village in the Democratic Republic of the Congo. *Malar J* 2016;15:431.
18. Rowland M, Webster J, Saleh P, Chandramohan D, Freeman T, Pearcy B, Durrani N, Rab A, Mohammed N. Prevention of malaria in Afghanistan through social marketing of insecticide-treated nets: evaluation of coverage and effectivenss by cross-sectional surveys and passive surveillance. *Trop Med Int Health* 2002;7(10):813-22.
19. Zhao Y, Zeng J, Zhao Y, Liu Q, He Y, Zhang J, Yang Z, Fan Q, Wang Q, Cui L, Cao Y. Risk factors for asymptomatic malaria infections from seasonal cross-sectional surveys along the China-Myanmar border. *Malar J* 2018;17:247.

For peer review only - http://bmjopen.bmj.com/site/about/guidelines.xhtml

20. Wahid S, Stresman GH, Kamal SS, Sepulveda N, Kleinschmidt I, Bousema T, Drakeley C. Heterogenous malaria transmission in long-term Afghan refugee populations: a cross-sectional study in five refugee camps in northern Pakistan. *Malar J* 2016;15:245.
21. Charlwood JD, Qassim M, Elnsur EI, *et al.* The impact of indoor residual spraying with malathion on malaria in refugee camps in eastern Sudan. *Acta Trop* 2001;80(1):1-8.
22. Rowland M, Nosten F. Malaria epidemiology and control in refugee camps and complex emergencies. *Ann Trop Med Parasit* 2001;95(8):741-54.
23. Rowland M, Hewitt S, Durrani N. Prevalence of malaria in Afghan refugee villages in Pakistan sprayed with lambdacyhalothrin or malathion. *Trans R Soc Trop Med Hyg* 1994;88:378-79.
24. Rowland M, Hewitt S, Durrani N, Bano N, Wirtz R. Transmission and control of vivax malaria in Afghan refugee settlements in Pakistan. *Trans R Soc Trop Med Hyg* 1997;91:252-55.
25. Rowland, M, Hewitt S, Durrani N, *et al.* Sustainability of pyrethroid-impregnated bednets for malaria control in Afghan communities. *Bull World Health Organ* 1997;75(1):23-29.
26. World Health Organization 2012. Global plan for insecticide resistance management in malaria vectors. Geneva, Switzerland: World Health Organization 2012.
27. Bouma MJ, Parvez SD, Nesbit R, *et al.* Malaria control using permethrin applied to tents of nomadic Afghan refugees in northern Pakistan. *Bull World Health Organ* 1996;74(4):413-21.
28. Graham K, Mohammad N, Rehman H, *et al.* Insecticide-treated plastic tarpaulins for control of malaria vectors in refugee camps. *Med Vet Entomol* 2002;16:404-08.
29. Graham K, Rehman H, Ahmad M, *et al.* Tents pre-treated with insecticide for malaria control in refugee camps: an entomological evaluation. *Malaria J* 2004;3:25.
30. Burns M, Rowland M, N'Guessan R, *et al.* Insecticide-treated plastic sheeting for emergency malaria prevention and shelter among displaced populations: an observational cohort study in a refugee setting in Sierra Leone. *Am J Trop Med Hyg* 2012;87(2):242-50.
31. Graham K, Mohammad N, Rehman H, *et al.* Comparison of three pyrethroid treatments of top-sheets for malaria control in emergencies: entomological and user acceptance studies in an Afghan refugee camp in Pakistan. *Med Vet Entomol* 2002;16:199-206.
32. Rowland M, Durrani N, Hewitt S, *et al.* Permethrin-treated chaddars and top-sheets: appropriate technology for protection against malaria in Afghanistan and other complex emergencies. *Trans R Soc Trop Med Hyg* 1999;93(5):465-72.
33. Macintyre K, Sosler S, Letipila F, *et al.* A new tool for malaria prevention?: results of a trial of permethrin-impregnated bedsheets (*shukas*) in an area of unstable transmission. *Int J Epidemiol* 2002;32:157-60.
34. Kimani EW, Vulule JM, Kuria IW, *et al.* Use of insecticide-treated clothes for personal protection against malaria: a community trial. *Malaria J* 2006;5:63.
35. Sochantha T, Van Bortel W, Savonnaroth S, *et al.* Personal protection by long-lasting insecticidal hammocks against the bites of forest malaria vectors. *Trop Med Int Health* 2010;15(3):336-41.
36. Sharma SK, Upadhyay AK, Haque MA, *et al.* Field evaluation of ZeroFly--an insecticide incorporated plastic sheeting against malaria vectors and its impact on malaria transmission in tribal areas of northern Orissa. *Indian J Med Res* 2009;130(4):458-66.
37. Messenger LA, Miller NP, Adeogun AO, *et al.* The development of insecticide-treated durable wall lining for malaria control: insights from rural and urban populations in Angola and Nigeria. *Malaria J* 2012;11:332.

For peer review only

For peer review only - http://bmjopen.bmj.com/site/about/guidelines.xhtml

38. Kitau J, Oxborough R, Kaye A, *et al.* Laboratory and experimental hut evaluation of a long-lasting insecticide treated blanket for protection against mosquitoes. *Parasit Vectors* 2014;7:129.
39. Reyburn H, Ashford R, Mohsen M, *et al.* A randomised controlled trial of insecticide-treated bednets and chaddars or top-sheets, and residual spraying of interior rooms for the prevention of cutaneous leishmaniasis in Kabul, Afghanistan. *Trans R Soc Trop Med Hyg* 2000;94:361-366.
40. Rowland M, Downey G, Rab A, *et al.* DEET mosquito repellent provides personal protection against malaria: a household randomized trial in an Afghan refugee camp in Pakistan. *Trop Med Int Health* 2004;9(3):335-42.
41. Rowland M, Freeman T, Downey G, *et al.* DEET mosquito repellent sold through social marketing provides personal protection against malaria in an area of all-night mosquito biting and partial coverage of insecticide-treated nets: a case-control study of effectiveness. *Trop Med Int Health* 2004;9(3):343-50.
42. McGready R, Simpson JA, Htway M, White NJ, Nosten F, Lindsay SW. A double-blind randomized therapeutic trial of insect repellents for the prevention of malaria in pregnancy. *Trans R Soc Trop Med Hyg* 2001;95:137-38.
43. Rowland M, Durrani N, Kenward M, *et al.* Control of malaria in Pakistan by applying deltamethrin insecticide to cattle: a community-randomised trial. *Lancet* 2001;357(9271):1837-41.
44. World Health Organization 2019.Global Malaria Programme. Guidelines for Malaria Vector Control.
45. World Health Organization 2020. List of WHO prequalified vector control products www.who.int/pq-vector-control/prequalified-lists/PrequalifiedProducts27January2020.pdf?ua=1. Accessed June 17th 2020.
46. Maia MF, Kliner M, Richardson M, *et al.* Mosquito repellents for malaria prevention. *Cochrane Database Syst Rev* 2018;2.
47. Furnival-Adams J, Olanga EA, Napier M, *et al.* Housing interventions for preventing malaria. *Cochrane Database Syst Rev* 2019;8.
48. Higgins JPT, Thomas J, Chandler J, *et al.* eds. *Cochrane Handbook for Systematic Reviews of Interventions* version 6.0 (updated July 2019). Cochrane, 2019. Available from www.training.cochrane.org/handbook.
49. Shea B, Robertson J, Peterson J, Welch V, Losos M. The Newcastle-Ottawa Scale (NOS) for Assessing the Quality of Nonrandomized Studies in Meta- Analysis Bias and Confounding Newcastle-Ottowa Scale. 2012. http://www.evidencebasedpublichealth.de/download/Newcastle_Ottowa_Scale_Pope_Bruce.pdf Accessed June 7th 2021.
50. Akl EA, Johnston BC, Alonso-Coello P, *et al.* Addressing dichotomous data for participants excluded from trial analysis: a guide for systematic reviewers. *PLoS One* 2013;8(2):e57132.
51. Nordic Cochrane Centre, The Cochrane Collaboration. Review Manager 5 (RevMan 5). Version 5.3. Copenhagen: Nordic Cochrane Centre, The Cochrane Collaboration, 2014.
52. Guyatt GH, Oxman AD, Schunemann HJ, *et al.* GRADE guidelines: a new series of articles in the Journal of Clinical Epidemiology. *J Clin Epidemiol* 2011;64(4):380-2.
53. Balshem H, Helfand M, Schunemann HJ, *et al.* GRADE guidelines: 3. Rating the quality of evidence. *J Clin Epidemiol* 2011;64(4):401-6.
54. Shamseer L, Moher D, Clarke M, *et al.* Preferred reporting items for systematic review and meta-analysis protocols (PRISMA-P) 2015: elaboration and explanation. *BMJ* 2015;349:g7647.

For peer review only

753 **Table 1.** Key factors influencing choice of vector control intervention(s) during humanitarian
754 emergencies.
755

Key Factors	Sub-Factors
Malaria epidemiology	Level of endemicity; local transmission dynamics and stability; circulating *Plasmodium* species and proportions of co-infection; level of drug resistance; asymptomatic infections
Human population composition and behaviour	Population origin and size; displacement dynamics and mobility; level of prior malaria exposure; proportion of vulnerable individuals (for example, pregnant women, children and the elderly); age and sex distributions; sleeping location and night-time behaviour; knowledge of malaria; education; healthcare and treatment seeking behaviour; prevalence of comorbidities (for example, malnutrition, anaemia, HIV); gender mobility and work practices
Vector species distribution and behaviour	Local vector species composition; vector seasonality; host preference (anthropophillic/zoophillic); feeding time and location (early/late biting and indoor/outdoor biting); resting location (indoor/outdoor resting); level of insecticide resistance; flight range; breeding site preference
Emergency setting and infrastructure	Type of shelter or housing available (for example, *ad-hoc* refuse materials, plastic sheeting, tents, more permanent housing); road access and condition; camp or community organisation and layout; prior and present vector control interventions or programmes; timing of rainy seasons; potential to impact other vector-borne diseases (such as in areas of sympatric leishmaniasis) and nuisance insects (for example, lice, fleas, ticks, biting flies, bed bugs)

756
757
758
759
760
761
762
763
764
765
766

For peer review only - http://bmjopen.bmj.com/site/about/guidelines.xhtml

767 **Table 2.** Types of intervention to be included in this review.

Intervention Category	Examples of Intervention	Control*
Insecticide-treated temporary housing materials	Plastic sheeting; tarpaulins; tents	Untreated temporary housing material
Insecticide-treated bedding materials	Sheets; blankets; chaddars; top sheets	Untreated bedding material
Insecticide-treated wall linings	Plastic woven or non-woven mesh material used to cover housing structure/shelters	Untreated wall lining
Insecticide-treated clothing	Military uniforms; occupational clothing	Untreated clothing
Insecticide-treated curtains	Netted curtain or window material	Untreated curtains
Insecticide treatment of livestock	Cattle dipping; use of endectocides	No cattle dipping/endectocide use
Personal repellents	Spatial repellents; topical repellents; insecticidal soaps	No spatial repellent
Bed nets	Long-lasting insecticidal nets (LLINs); insecticide-treated nets (ITNs); untreated nets (UTNs)	Untreated bed nets (when compared to insecticidal counterparts)
Indoor residual spraying (IRS)	Chemical treatment of housing structure/shelters	No chemical treatment of housing structures/shelters
Larval source management (LSM)	Chemical or biological treatment of breeding sites; habitat modification or manipulation	No insecticidal or mechanical control

768 *Studies where the control group comprises no other insecticidal malaria vector control
769 intervention will also be considered. Use of other vector control tools (in addition to the
770 intervention under evaluation) must be balanced between study arms.
771
772
773
774

For peer review only

For peer review only

For peer review only - http://bmjopen.bmj.com/site/about/guidelines.xhtml

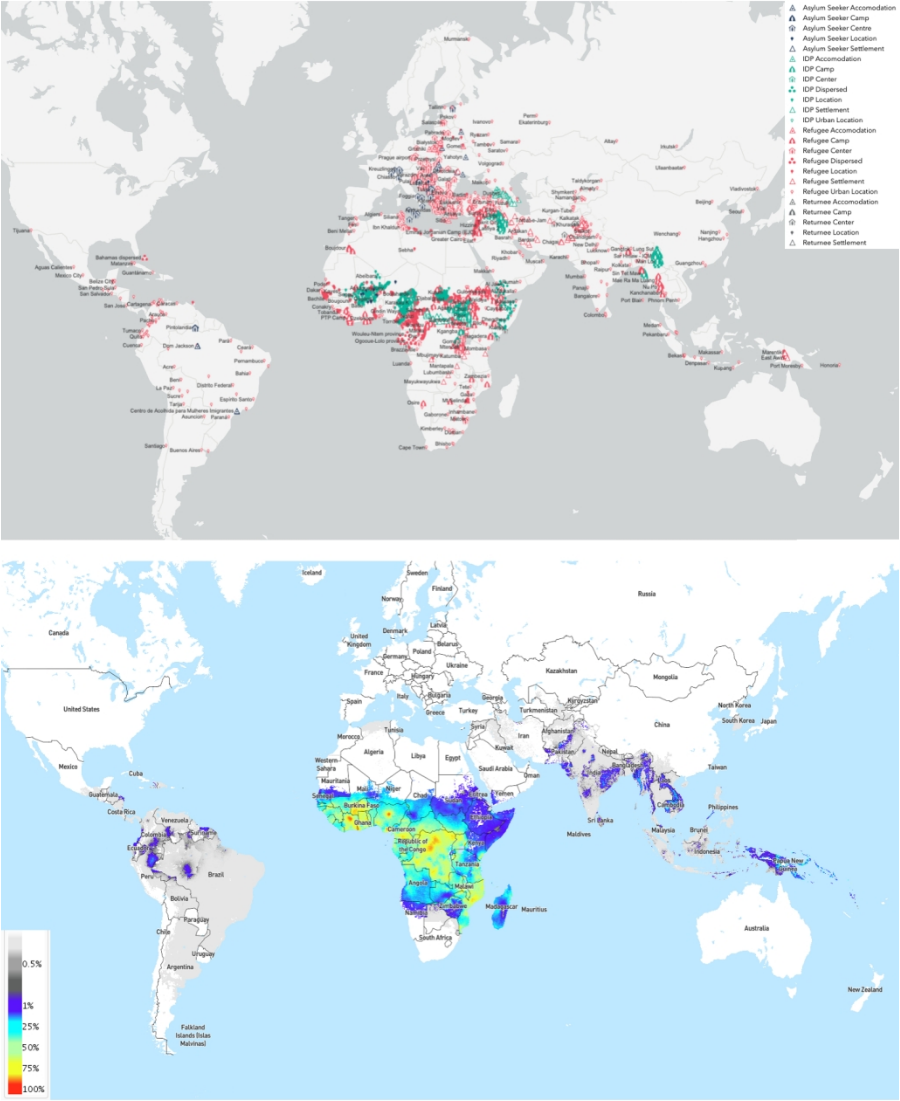

Figure 1. Left: Global map of internally displaced people (green) and refugee (red) populations, 2020. The UNHCR currently estimates there are 79.5 million forcibly displaced people, including 45.7 million internally displaced people, 26 million refugees, 4.2 million asylum-seekers and 3.6 million Venezuelans displaced abroad. Right: Global map of Plasmodium falciparum parasite rate in children aged 2-10 years old, 2000-2017.

299x400mm (127 x 127 DPI)

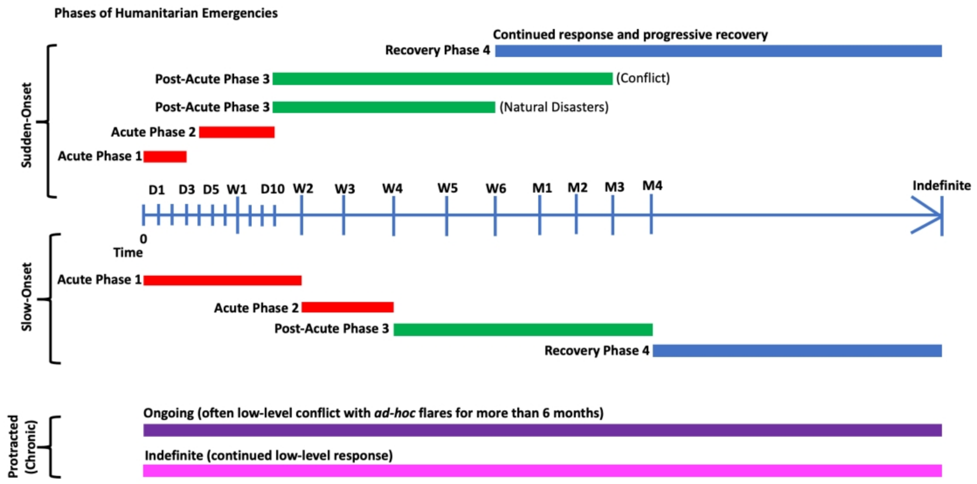

Figure 2. Phases of sudden-onset, slow-onset or protracted (chronic) humanitarian emergencies. Time is indicated in days (D), weeks (W) and months (M). Adapted from 3.

338x190mm (150 x 150 DPI)

PRISMA-P 2015 Checklist

This checklist has been adapted for use with protocol submissions to *Systematic Reviews* from Table 3 in Moher D et al: Preferred reporting items for systematic review and meta-analysis protocols (PRISMA-P) 2015 statement. *Systematic Reviews* 2015 **4**:1

Section/topic	#	Checklist item	Information reported Yes	Information reported No	Line number(s)
**ADMINISTRATIVE INFORMATION**					
**Title**					
Identification	1a	Identify the report as a protocol of a systematic review	X		1-2
Update	1b	If the protocol is for an update of a previous systematic review, identify as such		X	
**Registration**	2	If registered, provide the name of the registry (e.g., PROSPERO) and registration number in the Abstract	X		42
**Authors**					
Contact	3a	Provide name, institutional affiliation, and e-mail address of all protocol authors; provide physical mailing address of corresponding author	X		1-12
Contributions	3b	Describe contributions of protocol authors and identify the guarantor of the review	X		556-558
**Amendments**	4	If the protocol represents an amendment of a previously completed or published protocol, identify as such and list changes; otherwise, state plan for documenting important protocol amendments	X		549-551
**Support**					
Sources	5a	Indicate sources of financial or other support for the review	X		560-565
Sponsor	5b	Provide name for the review funder and/or sponsor	X		560-565
Role of sponsor/funder	5c	Describe roles of funder(s), sponsor(s), and/or institution(s), if any, in developing the protocol	X		568-571
**INTRODUCTION**					
**Rationale**	6	Describe the rationale for the review in the context of what is already known	X		61-178
**Objectives**	7	Provide an explicit statement of the question(s) the review will address with reference to participants, interventions, comparators, and outcomes (PICO)	X		180-195
**METHODS**					

BioMed Central The Open Access Publisher

Section/topic	#	Checklist item	Information reported		Line number(s)
			Yes	No	
**Eligibility criteria**	8	Specify the study characteristics (e.g., PICO, study design, setting, time frame) and report characteristics (e.g., years considered, language, publication status) to be used as criteria for eligibility for the review	X	☐	198-302; 342-395
**Information sources**	9	Describe all intended information sources (e.g., electronic databases, contact with study authors, trial registers, or other grey literature sources) with planned dates of coverage	X	☐	310-319
**Search strategy**	10	Present draft of search strategy to be used for at least one electronic database, including planned limits, such that it could be repeated	☐	X	
STUDY RECORDS					
Data management	11a	Describe the mechanism(s) that will be used to manage records and data throughout the review	X	☐	344-348
Selection process	11b	State the process that will be used for selecting studies (e.g., two independent reviewers) through each phase of the review (i.e., screening, eligibility, and inclusion in meta-analysis)	X	☐	330-340
Data collection process	11c	Describe planned method of extracting data from reports (e.g., piloting forms, done independently, in duplicate), any processes for obtaining and confirming data from investigators	X	☐	344-349
**Data items**	12	List and define all variables for which data will be sought (e.g., PICO items, funding sources), any pre-planned data assumptions and simplifications	X	☐	350-395
**Outcomes and prioritization**	13	List and define all outcomes for which data will be sought, including prioritization of main and additional outcomes, with rationale	X	☐	234-302
**Risk of bias in individual studies**	14	Describe anticipated methods for assessing risk of bias of individual studies, including whether this will be done at the outcome or study level, or both; state how this information will be used in data synthesis	X	☐	426-442
DATA					
**Synthesis**	15a	Describe criteria under which study data will be quantitatively synthesized	X	☐	446-452
	15b	If data are appropriate for quantitative synthesis, describe planned summary measures, methods of handling data, and methods of combining data from studies, including any planned exploration of consistency (e.g., I^2, Kendall's tau)	X	☐	482-491
	15c	Describe any proposed additional analyses (e.g., sensitivity or subgroup analyses, meta-regression)	X	☐	523-541
	15d	If quantitative synthesis is not appropriate, describe the type of summary planned	☐	X	
**Meta-bias(es)**	16	Specify any planned assessment of meta-bias(es) (e.g., publication bias across studies, selective reporting within studies)	X	☐	493-497
**Confidence in cumulative evidence**	17	Describe how the strength of the body of evidence will be assessed (e.g., GRADE)	X	☐	506-520

For peer review only

For peer review only - http://bmjopen.bmj.com/site/about/guidelines.xhtml

BioMed Central The Open Access Publisher
